# Assessment of Task-Specific Gait and Balance Deficits in Chronic Low Back Pain Using Multi-Sensor Inertial Measurement Units †

**DOI:** 10.3390/s26196182

**Published:** 2026-09-29

**Authors:** Yen-Chang Chien, Lik-Kang Koo, Chia-Wei Chang, Chien-Cheng Chen, Szu-Fu Chen, Fu-Cheng Wang

**Affiliations:** 1 Department of Physical Medicine and Rehabilitation, Cheng Hsin General Hospital, Taipei 112, Taiwan; cute29554416@gmail.com (Y.-C.C.); josephlkkoo@gmail.com (L.-K.K.); braddom.chen@gmail.com (C.-C.C.); 2 Department of Mechanical Engineering, National Taiwan University, Taipei 106, Taiwan; r12522849@ntu.edu.tw; 3 Graduate Institute of Gerontology and Health Care Management, Chang Gung University of Science and Technology, Taoyuan 333, Taiwan; 4 Department of Physiology and Biophysics, National Defense Medical University, Taipei 114, Taiwan

**Keywords:** chronic low back pain, gait, balance, inertial measurement unit, angular velocities

## Abstract

Chronic low back pain (cLBP) compromises daily function, but associated balance deficits remain difficult to quantify using traditional scales. We recruited 50 participants with CLBP (age: 65.68 ± 11.84) and 50 age-matched controls (age: 65.26 ± 10.73) wearing multiple inertial measurement units (IMUs) to evaluate kinematics: average absolute angular velocity (Jω) and linear acceleration (Jα) across static (single-leg stance), perturbed (balance board), locomotor (walking, tandem gait), and transitional (five times sit-to-stand) tasks. Group differences were assessed using multivariate analysis of variance and Bonferroni-adjusted univariate tests. During the balance board test,  Jω was significantly higher at the chest (p  = 0.027) and hip (p  = 0.003) in the CLBP group. Conversely, during the sit-to-stand tests, the CLBP group showed significantly lower Jα and  Jω (*p* < 0.0001) at the hip. The CLBP group exhibited globally lower  Jω (p  < 0.01) during normal walking, while the ${Amp}_{PR}$ (p  = 0.001) was exclusively reduced during tandem gait. These results suggest that multi-sensor IMU-based assessments provide a sensitive tool for detecting subtle, task-specific balance deficits in CLBP, indicating that rehabilitation should target rotational mobility and reactive control rather than core stiffening alone. Larger-scale and longitudinal research is still required to determine their clinical utility.

## 1. Introduction

Chronic low back pain (CLBP) is a major contributor to disability worldwide [1], while profoundly compromising gait, balance, and daily activities [2,3]. Generally, CLBP is established as localized lumbosacral pain that continues for more than three months [4]. Clinical management focuses primarily on pain reduction. However, research suggests that CLBP arises from multifactorial interactions between biomechanical alterations, neuromuscular deficits, and kinesiophobia [5,6,7]. The established literature documents that individuals suffering from CLBP often adopt a “protective trunk stiffening strategy” to manage perceived spinal instability and minimize pain [8]. This adaptation may increase trunk muscle co-activation, restrict flexible postural control and heighten susceptibility to repeated flare-ups [9].

In practical healthcare applications, functional performance is assessed using categorical or ordinal scales, such as the timed up-and-go (TUG) test, single-leg stance test (SLST), and five times sit-to-stand (FTSS) test. These scales provide limited quantitative resolution and may fail to detect subtle coordination impairments [10]. Furthermore, a “ceiling effect” that can mask early-stage motor impairments is frequently observed in higher-functioning patients [11]. Although laboratory-grade systems, including optoelectronic motion systems, surface electromyography and force plates, yield high-quality kinematic measures, their cost and complex operational requirements make them impractical for large-scale clinical screening [12,13].

To overcome these practical limitations, wearable inertial measurement units (IMUs) serve as a cost-effective and readily implementable substitute for motion capture. Systematic reviews have affirmed the measurement fidelity and consistency of IMUs for lumbar kinematic analysis [14]. However, despite their clinical accessibility, current IMU-based studies have not yet fully integrated comprehensive functional assessments of CLBP, owing to several notable methodological limitations. First, previous research has largely captured isolated movements rather than the full kinetic chain dynamics. Most studies limited the evaluation of intersegmental coordination and compensatory strategies across the broader kinetic chain [15,16,17,18]. Second, assessment protocols are typically restricted to static or dynamic conditions alone [15,19,20]. For example, although some studies have associated CLBP with increased sway during static postural control [21,22], they rarely extended their kinematic analysis to perturbation conditions, leaving reactive balance mechanisms largely unexplored. Considering that static and dynamic stability exhibit distinct patterns of deterioration during disease progression or aging [23], they are likely governed by distinct sensorimotor processes that warrant concurrent evaluation [24]. Furthermore, the interpretation of these isolated tests can be difficult when studies lack healthy control groups [16,21]. Finally, conventional metrics may lack the resolution required to detect subtle motor control deficits. Although diminished pelvic contribution [25] and altered movement speeds are frequently observed using standard parameters, such as mean velocity [21], peak-to-peak amplitude [26], and center of pressure [22], the literature presents conflicting findings regarding functional interventions. For instance, one meta-analysis found no significant dynamic balance improvements in patients with CLBP following proprioceptive neuromuscular facilitation [22], whereas a more recent meta-analysis reported positive effects of stabilization exercises [27]. These discrepancies highlight the need for highly sensitive kinematic indicators. Moreover, traditional functional tests often mask phase-specific impairments; for example, kinematic alterations during the FTSS differ significantly between the rising and lowering phases. Therefore, the major gap is that the task-specific effects of CLBP on multisegment balance and gait control remain insufficiently characterized using wearable IMU-based metrics across static, dynamic, and transitional conditions.

There are two key metrics from the IMU triaxial vector signals: average absolute angular velocity (Jω) and average absolute linear acceleration (Jα) were introduced to record kinematic data. These metrics are based on the mean amplitude deviation (MAD) method, which has been validated to reduce errors from sensor placement orientation and maintain high accuracy in quantifying both the intensity and variability of movement [28,29].

These indices have been implemented in previous studies and verified as sensitive markers for characterizing dynamic shifts in movement [23,30,31]. Importantly, prior research [23,30,31] has demonstrated that Jω and Jα reflect different aspects of movement depending on the context: in postural maintenance tasks, elevated values indicate greater instability due to increased corrective movements, whereas in functional dynamic tasks, higher values are associated with more effective and efficient motor performance. During postural maintenance tasks, higher values of Jω and Jα represent greater disturbance in maintaining equilibrium through frequent angular and linear adjustments, while in functional dynamic tasks, higher values of those indices indicate better motor efficiency. Given that CLBP patients often exhibit both balance deficits and increased trunk stiffness, we hypothesized that individuals with CLBP would show higher Jω and Jα during stationary balance assessments, but lower Jω and Jα  during dynamic tasks and gait-related transitions than healthy controls.

In the gait assessment, pelvic rotation amplitude (${Amp}_{PR}$) was used to evaluate locomotor efficiency. During gait initiation, pelvic rotation facilitates stride lengthening and energy conservation [32,33]. Clinically, patients with CLBP may involuntarily avoid excessive trunk motion to reduce pain [34], and the trunk-stiffening pattern is more obvious during a challenging postural control test [35]. Consequently, we further hypothesized that the CLBP group would exhibit a reduced pelvic rotation amplitude compared with healthy individuals during walking tasks, reflecting a more constrained and less efficient locomotor pattern.

Our study attempted to extend the previous literature through three distinct aspects: (i) systematic evaluation of the full kinetic chain synergies and phase-specific analysis, deploying a multiple IMU setup to observe intersegmental compensations across the body (chest, lumbar, hip, lower limbs) while isolating movement phases (e.g., sit-to-stand rising vs. stand-to-sit lowering); (ii) integration of cross-modal functional testing, combining static postural maintenance without perturbation (SLST), self-induced balance board perturbations (balance board test), locomotor challenges (tandem gait), and dynamic functional transitions (FTSS) to explore the full components of sensorimotor performance; and (iii) employing Jω, Jα as parameters to capture kinematic deviations that remain undetected by standard range of motion measures and differentiate rotational velocity from linear acceleration. Accordingly, our study projected that individuals suffering from CLBP would manifest task-dependent movement divergences across multi-segment kinematics, characterized by higher Jω and Jα during static balance tasks, lower Jω and Jα during dynamic tasks, and reduced pelvic rotation amplitude (${Amp}_{PR}$) during tandem gait compared with healthy controls. By testing these hypotheses, this study aimed to provide a more sensitive and objective characterization of CLBP-related balance and locomotor impairments, thereby enabling movement-oriented clinical assessments and interventions.

## 2. Materials and Methods

### 2.1. Experimental Design and Ethical Approval

This cross-sectional study received ethical clearance from the Institutional Review Board of Cheng Hsin General Hospital, Taiwan (Protocol Code: (1174)114-32), and all procedures conformed to the Declaration of Helsinki. Prior to enrollment, all participants granted written permission to use their personal data recorded in this study. It was also clear that the participants had the right to withdraw at any stage. After recruitment, we collected demographic and clinical data to confirm eligibility for this study. To assess different aspects of motor function, the experimental protocol across four functional domains was adapted from established clinical scales (such as the Berg Balance Scale) and international clinical guidelines, including the APTA CPGs linked to ICF [36], NIH Task Force Standards [37], and European Guidelines for CLBP [38]. Selected for their high clinical accessibility and short administration time, these standardized tasks were combined with wearable IMUs to provide objective kinematic profiles of multi-segment body movement.

We sequentially administered the balance board test (BBT), walking tests under normal and tandem gait, SLST, and FTSS. Each test was performed twice, and rest between trials was allowed as needed. The best performance was selected in further analysis.

**Figure 1:**
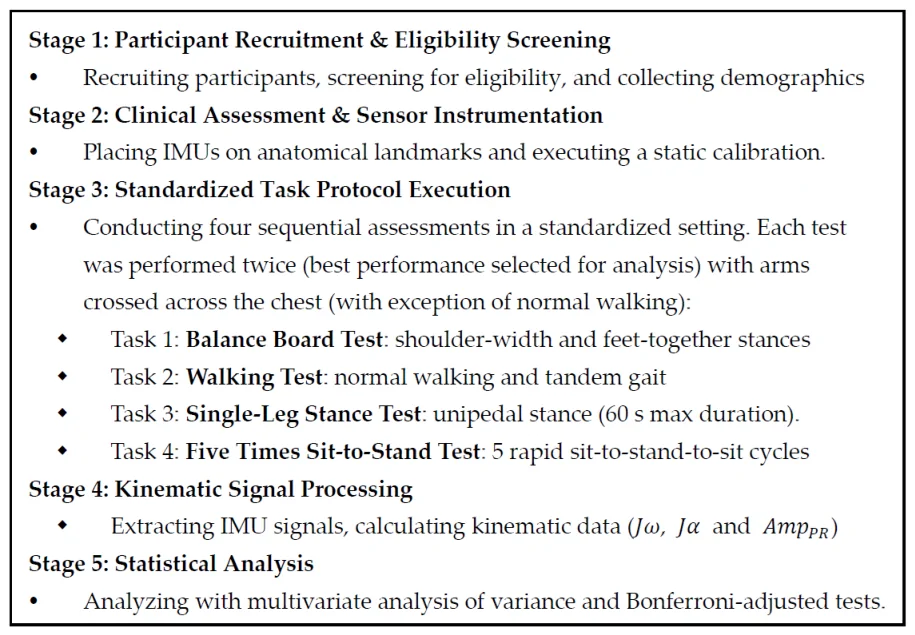
Experimental flow diagram of the entire experimental process.

An experimental flow diagram is presented in Figure 1. All testing procedures were conducted by trained personnel following standardized safety and instruction guidelines.

### 2.2. Participants

CLBP and 50 age-matched asymptomatic controls. Patients within the CLBP group presented with continuous low back pain persisting for at least three months. To ensure safety and outcomes reflecting postural control rather than acute pain effects, we included only participants who reported mild (1–4) or moderate (5–6) pain on the Numeric Pain Rating Scale (NPRS). The control group was required to be free of back pain or related therapeutic interventions for at least 6 months before testing. The participants were adults (aged > 20 years), cognitively intact, and able to walk 30 m unaided. To ensure data validity, we excluded individuals with prior spinal or lower limb surgery, neurodegenerative disorders, cognitive impairment, pregnancy, and those at a high risk of falling.

Physical disability and functional limitations were quantified using the Roland–Morris Disability Questionnaire (RMDQ) [39]. The RMDQ was selected for its high sensitivity in assessing patients with mild-to-moderate disability and consists of 24 items designed to evaluate physical functions compromised by low back pain. The validated traditional Chinese version was used to ensure accuracy in the local population [40].

We recruited patients with CLBP from a physical medicine and rehabilitation outpatient clinic. Healthy adults from the community were recruited through community centers and advertisements and matched by age as controls.

The healthy control group (mean age: 65.26 ± 10.73 years) comprised independent, community-dwelling adults with age-appropriate physical performance. All control participants met the defined study exclusion criteria. Functional mobility was shown by a mean FTSS time of 9.5 ± 2.0 s. When compared with international population reference values for healthy adults aged 60–69 years (9–11 s) [41], this FTSS time falls within the 50th to 60th percentiles, confirming that the control group reflects typical, non-athletic community-dwelling adults.

To ensure an adequate sample size for statistical power, a power analysis was conducted in G*Power 3.1.9.7 (Heinrich Heine University Düsseldorf, Germany) targeting a two-group contrast. The statistical parameters were set with a type I error rate of 0.05 (α = 0.05) and a target power of 0.80 (power = 0.80). The expected effect size (0.25–0.40, medium-to-large) was estimated based on the magnitude of group differences in Jω and Jα observed across static, dynamic, and transitional tasks in our previous IMU studies, which consistently demonstrated moderate-to-large between-group discrepancies in these kinematic parameters [23,30,42,43]. The required total sample size was estimated to be between 52 and 128 participants (26–64 per group). To account for potential drop-out rate of 10%, we enrolled 50 participants per group (total N = 100), ensuring adequate statistical power to detect kinematic differences.

### 2.3. Kinematic Instrumentation

Segmental motion capture relied on the Opal IMU system (APDM Wearable Technologies, Portland, OR, USA) to acquire kinematic metrics [44]. The system consists of synchronized wireless units that integrate triaxial gyroscopes, accelerometers, and magnetometers. To ensure high-resolution data, the sensors were equipped with built-in filters operating across a sampling frequency window of 20 to 128 Hz. In this study, the IMU system was set to the highest sampling rate (128 Hz) to achieve sufficient sensitivity for detecting subtle coordination deficits, as previously reported [45]. Additional details regarding the Opal IMU system are provided in Appendix A.

**Figure 2:**
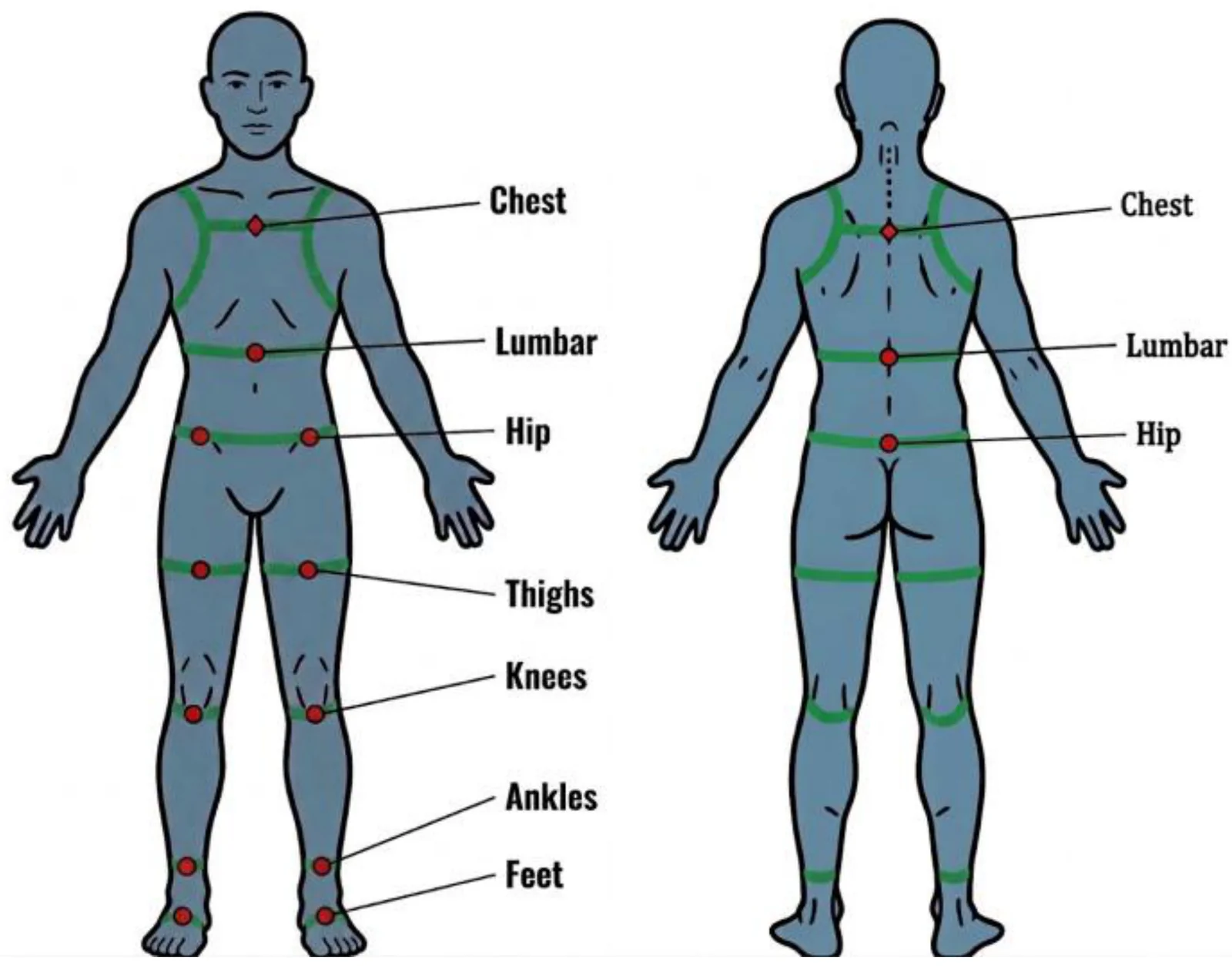
Schematic diagram showing IMU placements. Sensors (red dots) are positioned at the chest (scapular spine), lumbar region (L2–L3), hip (posterior superior iliac spine, PSIS), thighs (midpoint between hip and knee joints), knees (inferior patellar pole), ankles (proximal border of lateral malleolus), and dorsal feet (metatarsals). Green lines indicate the wearing configuration.

According to the requirements of different functional tests, IMUs were secured to designated anatomical landmarks, adhering to previous research [23,30,46,47]. In Figure 2, the green lines indicate the wearing configuration, and the red dots represent the sensor locations. Skin-motion artifacts were minimized by securing the sensors to the skin with tape or straps over the designated anatomical landmarks. The positions of IMU are listed in Table 1.

Before each trial, a static calibration procedure was performed to establish a consistent measurement origin for each participant. This procedure ensured that the angular reference frames were standardized across the participants. Specifically, the sensor orientations were aligned with the anatomical segments to maintain consistency in the measurements across participants.

### 2.4. Parameters of Kinematic Data

To objectively quantify balance control and gait performance, we focused on three biomechanical indices derived from the IMU signals: the average absolute angular velocity (Jω), the average absolute linear acceleration (Jα), and the pelvic rotation amplitude (${Amp}_{PR}$). These indices were selected because of their high reliability and their ability to detect subtle variations in balance control in our series of IMU-based studies [23,30,31,43].

We defined the average absolute angular velocity (Jω) and the average absolute linear acceleration (Jα) for each segment k using the following formula:

(1) $$J_{\omega }^{k}=\frac{1}{N}\sum_{i=1}^{N}\left|\omega^{k}(i)\right|=\frac{1}{N}\sum_{i=1}^{N}{\left((\omega_{x}^{k}(i){)}^{2}+(\omega_{y}^{k}(i){)}^{2}+(\omega_{z}^{k}(i){)}^{2}\right)}^{1/2}and$$

(2) $$J_{\alpha }^{k}=\frac{1}{N}\sum_{i=1}^{N}\left|\alpha^{k}(i)\right|=\frac{1}{N}\sum_{i=1}^{N}{\left((\alpha_{x}^{k}(i){)}^{2}+(\alpha_{y}^{k}(i){)}^{2}+(\alpha_{z}^{k}(i){)}^{2}\right)}^{1/2},$$

In these formulas, k∈{chest,lumbar,hip,thighs,knees,ankles,feet,balanceboard} designates the targeted body segment and N quantifies the total sample size. The components of $\omega_{x}^{k}\left(i\right),\omega_{y}^{k}\left(i\right),\omega_{z}^{k}\left(i\right),\alpha_{x}^{k}\left(i\right),\alpha_{y}^{k}\left(i\right),and\;\alpha_{z}^{k}(i)$ refer to the tri-axial angular velocity (rad/s) and linear acceleration (${m/s}^{2}$) measured at location k for time index i using the IMU. These metrics, $J_{\omega }^{k}\;and\;J_{\alpha }^{k}$, effectively quantify the mean intensity of rotational and linear kinematics, respectively. In our analysis, they serve as crucial indicators of both postural stability and motor control efficiency.

Jω  and Jα are grounded in the biomechanical principle of Mean Amplitude Deviation (MAD), which is commonly used in wearable devices [28,29]. We adopt a triaxial vector magnitude approach, which has been shown in the literature to effectively eliminate errors caused by sensor placement angles and to quantify movement variability.

In particular, the interpretation of Jω and Jα was opposite between stationary and dynamic tasks. When the values increase in the stationary tests, they reflect unstable compensatory movements. Conversely, when the values decrease in the dynamic tests, they reveal a protective trunk-stiffening pattern and pain-avoidance strategies.

Biomechanically, Jω and Jα were selected to provide distinct and complementary clinical insights depending on task demands. Jω quantifies rotational movement magnitude. In stationary perturbed tasks (such as the BBT), elevated Jω is expected to reflect excessive upper-body rotation and delayed corrective actions required to maintain balance, whereas during natural walking, reduced Jω is hypothesized to indicate protective trunk guarding aimed at minimizing spinal movement. In contrast, Jα measures linear translational acceleration, which is particularly responsive to dynamic weight transfers. During the FTSS, reduced Jα during the sit-to-stand rising phase is anticipated to isolate lumbopelvic weakness and reduced upward acceleration when rising against body weight.

A full gait cycle is delineated by three pivotal events: heel strike, toe-off, and mid-swing. The pelvic rotation amplitude (${Amp}_{PR}$) quantifies the total angular range of pelvic rotation across successive heel-strike events. The formula is as below:

(3) $${Amp}_{PR}\;=\;\theta_{Max}\;-\;\theta_{Min}$$

In this equation, $\theta_{Max}$ is the max pelvic angular displacement and $\theta_{Min}$ is the minimum pelvic angular displacement across a single stride. Because horizontal pelvic rotation is critical for efficient walking, evaluating *AmpPR* during normal and narrow-base tandem gait is expected to help detect restricted pelvic movement and protective guarding in CLBP.

These indices were computed across both postural maintenance tasks (SLST, BBT) and functional dynamic (walking, 5TSS) paradigms to construct a comprehensive balance profile. We aimed to delineate the distinct difference in postural control between the CLBP group and healthy controls.

### 2.5. Postural Maintenance Test Without Perturbations: Single-Leg Stance Test (SLST)

The single-leg stance test (SLST) was used to assess the static elements of balance control. To minimize potential order effects, the testing limb sequence was determined using a random number table. Initially, testing was conducted with subjects standing unshod on a rigid, level platform. They were instructed to stand on the designated limb while lifting their contralateral legs off the ground. During the test, subjects were instructed to maintain folded arms across their chest to eliminate upper-limb compensatory strategies. Additionally, a target was placed approximately 1 m ahead at eye level to standardize visual fixation and reduce postural instability caused by uncontrolled eye movements.

Each trial lasted for a maximum of 60 s and was conducted at least twice. A trial was terminated early if balance was lost, defined as the elevated foot touching the ground, the stance foot shifting from its original position, or the arms being uncrossed to regain stability. To ensure trial familiarity, subjects who failed to complete the SLST for 60 s were permitted up to a total of three attempts for each limb. A standardized rest period of 30 to 60 s was provided between successive trials to mitigate muscle fatigue. Finally, the trial with the maximum successful duration was extracted for final evaluation, as it best reflected the participant’s maximal balance capacity.

### 2.6. Postural Maintenance Test with Perturbations: Balance Board Test (BBT)

Wobble boards [48,49] and computerized dynamic posturography (CDP) [50] are commonly used to evaluate balance performance under perturbations; however, they have certain limitations. Wobble boards lack safety handrails and pose a danger to participants with a high risk of falling, whereas the substantial financial cost and spatial requirements of CDP systems largely confine their application. Consequently, a suspended balance board was used to ensure participant safety and accessibility.

**Figure 3:**
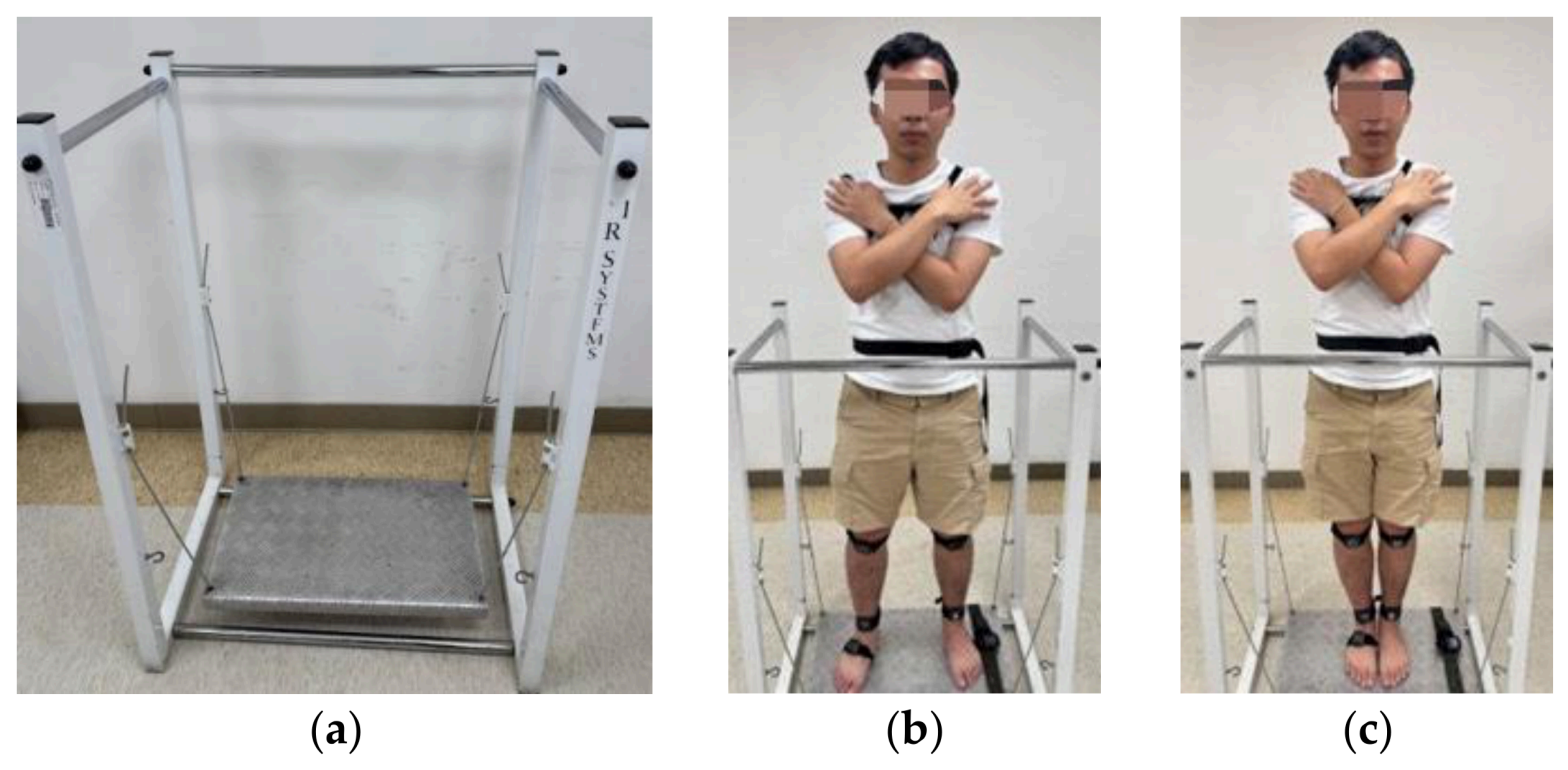
Overview of the balance board evaluation: (**a**) the suspended balance platform; (**b**) the shoulder-width stance; and (**c**) the narrow, feet-together stance.

The BBT was used to assess balance control under self-induced perturbations. This study used a suspended balance board system (Monitored Rehab Systems, Haarlem, Netherlands), which has been previously used to assess postural instability across various patient populations [23,30]. A 60 × 35 cm flat, firm platform was tethered via four high-tensile steel cables, creating a passively unstable foundation that allowed for small-angle sway (Figure 3a). Thus, this setup behaves like a pendulum undergoing simple harmonic oscillation. As the board swayed horizontally in response to the participants’ own weight shifts, it continuously generated self-induced perturbations. When transitioning from a standard floor to the moving surface, the participants must continuously adjust their motor control inputs to stabilize the board, thereby highlighting the dynamic components of postural maintenance.

Each trial lasted a maximum of 60 s and was repeated at least twice. During the test, the participants maintained an upright posture on the board with arms folded across the chest. Two conditions were sequentially evaluated: (i) foot shoulder width (Figure 3b) and (ii) feet together (Figure 3c). To prevent fatigue, standardized rest periods (30–60 s) were provided between trials, and the participants were permitted to grab the safety handrails at any point if they felt unstable. If a participant lost balance or stepped off the board, the trial was terminated, and the participant was allowed a total of three attempts per condition. Only the longest standing time across all attempts was used for further kinematic analysis.

### 2.7. Functional Dynamic Test: Walking Test

During the walking test, the participants walked forward and back barefoot along a 10 m linear path. To ensure safety, a research assistant accompanied each participant during all testing sessions as a physical guardian without interfering with their movement patterns. The participants were permitted to complete three practice trials before formal data collection.

The test was conducted using two gait patterns to assess dynamic stability. (i) Normal walking: participants walked with natural arm swings at their preferred, self-paced walking speed to capture unconstrained habitual gait mechanics. (ii) Tandem gait: participants executed a heel-to-toe progression with arms folded across the chest to eliminate upper-limb compensatory moments and isolate lumbopelvic balance control under increased mechanical demand. A trial was deemed invalid if the subject was unable to maintain heel-to-toe alignment, exhibited a marked lateral shift in the center of gravity, or required physical assistance to prevent falls.

Each test was conducted at least twice, and a standardized rest period of 30 to 60 s was provided between trials. For each task, the fastest successful trial was selected for kinematic analysis.

In addition to Jω and Jα, the pelvic rotation amplitude (AmpPR) was also measured to quantify lumbopelvic complex coordination during the gait cycle. To reduce artifacts from transitional events, only constant-speed strides from the middle portion of the walkway were analyzed.

### 2.8. Functional Dynamic Test: Five Times Sit-to-Stand Test (FTSS)

The five times sit-to-stand test (FTSS) was used to assess the functional transitional capacity and coordination of the kinetic chain. A seat height of a 40 cm armless chair was used for assessment. Testing was conducted with participants wearing standard footwear and arms folded across the chest. Participants performed the task by transitioning from a seated to a fully standing posture as rapidly as possible for five consecutive repetitions. The research assistant stabilized the back of the chair during the procedure. Each repetition required full hip and knee extension with a straightened trunk upon standing, followed by complete gluteal contact with the seat upon returning to the chair.

Each participant performed at least two formal trials with a standardized rest period of 30–60 s between attempts. Among the successful attempts, the trial yielding the shortest completion time was extracted for kinematic processing.

The kinematic inflection points from the IMU signals were used to segment the FTSS into distinct sit-to-stand and stand-to-sit phases. The analysis focused exclusively on the steady-state mid-phase of each movement, effectively filtering out the kinematic artifacts associated with the initiation and termination of each transition.

### 2.9. Data Processing and Statistical Methods

All statistical procedures were executed using IBM SPSS Statistics (Version 22.0; IBM Corp., Armonk, NY, USA). Because movements across different body segments are kinematically linked and interdependent, a Multivariate Analysis of Variance (MANOVA) was employed. This approach was selected to capture the interactions between segments and to prevent Type I error inflation. To account for multiple comparisons in univariate testing, Bonferroni-adjusted p-values were calculated to maintain a conservative sensitivity threshold. Continuous variable metrics are expressed as mean ± standard deviation (SD), with statistical significance established at p < 0.05.

## 3. Results

### 3.1. Demographic Data and Kinematic Parameters

We enrolled 100 eligible individuals for this study, including 50 patients with CLBP (mean ± SD [range]: 65.68 ± 11.84 years [26–83]) and 50 age-matched healthy controls (mean ± SD [range]: 65.26 ± 10.73 years [29–80]). Table 2 presents the demographic data in detail. The two cohorts demonstrated baseline comparability regarding age (p = 0.85) and body mass index (p  = 0.74). Furthermore, sex distribution was balanced between the CLBP group (36 females, 72%) and the control group (32 females, 64%).

Participants with CLBP reported an average pain chronicity of 6.94 ± 5.52 years (mean ± SD). The average pain intensity was 4.38 ± 1.47 points (mean ± SD), corresponding to a moderate level on the NPRS. To quantify functional impairment, the RMDQ was recorded, with the CLBP group presenting an average score of 7.14 ± 4.05 points (mean ± SD). This score classifies the CLBP group as having medium-to-high-level disability, suggesting greater fall susceptibility and functional risk [51]. Overall, these findings confirmed that the two groups were demographically similar, providing a comparable baseline for subsequent gait and balance analyses.

To facilitate clinical interpretation and provide baseline reference values, detailed descriptions of kinematic parameters (Jω, Jα, and ${Amp}_{PR}$) presented as mean ± standard deviation for both the healthy control cohort and the CLBP group across all static and dynamic tasks are provided in Table S1.

Main text figures focus on kinematic parameters and body segments with statistically significant group differences (p < 0.05). Complete dataset visualizations across all sensor locations for all functional tasks are provided in Figure S1.

### 3.2. Kinematic Data of the Single-Leg Stance Test

In the SLST, inter-group comparisons yielded no statistically meaningful variance in either rotational (Jω) or translational (Jα) kinematic indices across all measured anatomical landmarks (Figure S1). These findings indicate that patients with mild-to-moderate CLBP maintain adequate balance control during postural maintenance without perturbations. This lack of significant difference is likely due to the specific targeting of a mild-to-moderate CLBP cohort, which aligns with the known limited sensitivity of traditional static assessments.

Therefore, the SLST serves as a preliminary assessment to be contrasted with more rigorous dynamic tasks. Employing subsequent tasks that introduce mechanical instability is necessary to expose subtle coordination impairments that are typically undetectable during simple static tests.

### 3.3. Kinematic Data of the Balance Board Test

**Figure 4:**
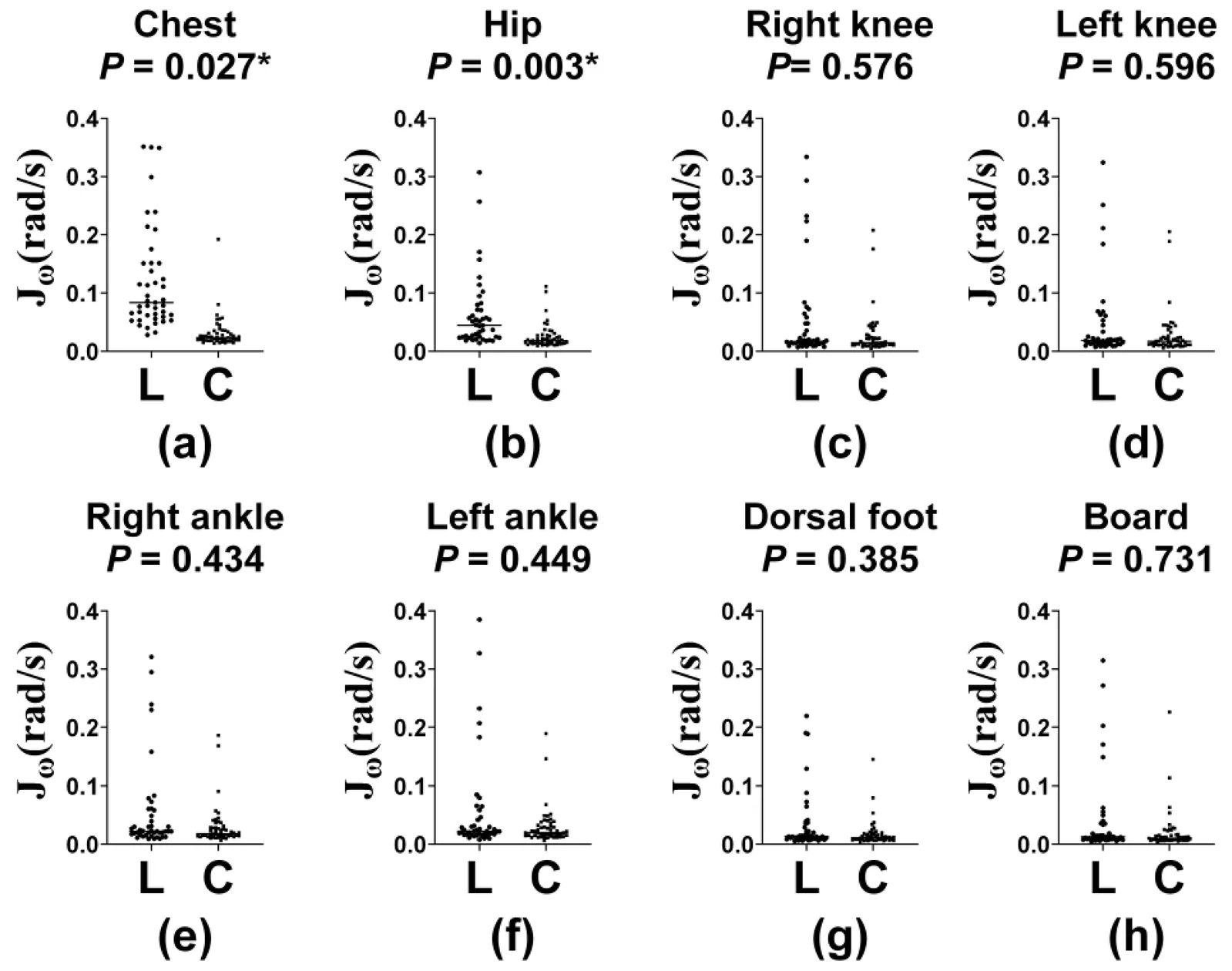
Segmental distribution of Jω (**a**–**h**) across body segments during the balance board stances with shoulder width. Data are presented as column scatter plots (horizontal bars indicate medians) comparing the chronic low back pain (L) and control (C) groups across body segments. Significantly elevated Jω values were observed at the chest (p = 0.027) and hip (p = 0.003) in the CLBP group, suggesting enhanced upper body and pelvic compensatory strategies during the shoulder-width BBT. * Bonferroni-corrected *p*-value < 0.05.

**Figure 5:**
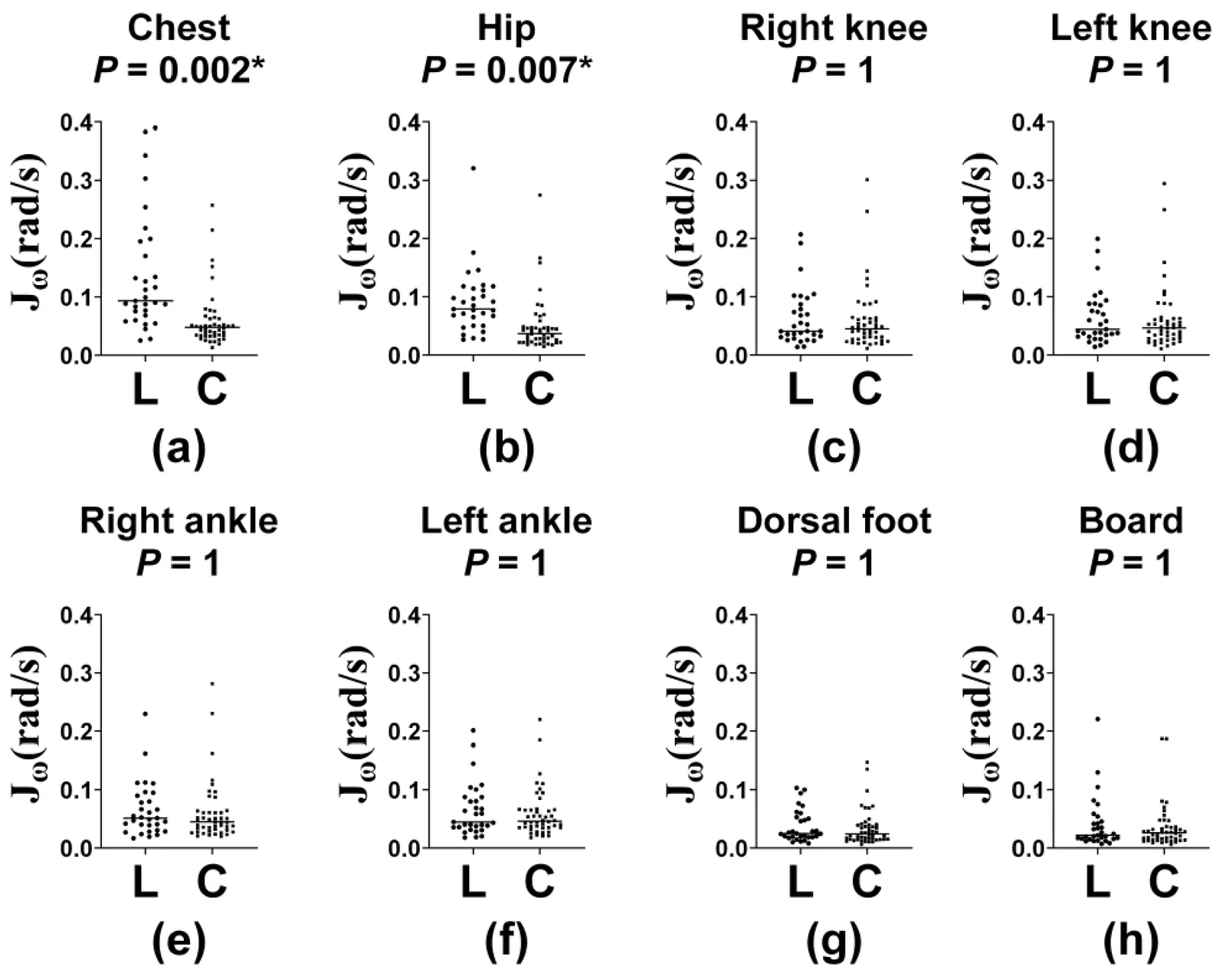
Segmental distribution of Jω(**a**–**h**) across body segments during the balance board stances with feet together. Data are presented as column scatter plots (horizontal bars indicate medians) comparing the chronic low back pain (L) and control (C) groups across body segments. Significantly elevated Jω  values were observed at the chest (*p* = 0.002) and hip (*p* = 0.007) in the CLBP group, suggesting enhanced upper body and pelvic compensatory strategies during the feet-together BBT. * Bonferroni-corrected *p*-value < 0.05.

In the BBT with shoulder-width stance (Figure 4), Jω demonstrated significantly higher values in the CLBP group at the chest (p = 0.027) and hip (p = 0.003). These results were consistent at the same locations (both p < 0.001) when the task demand advanced to the feet-together stance (Figure 5). Regarding Jα, no single body segment exhibited significant inter-group divergence (Figures S2 and S3).

These findings suggest that individuals with CLBP must make more pronounced angular adjustments to the trunk in response to postural disturbances. To compensate for limited lumbar mobility and fear avoidance of pain [8], they appear to increase upper body and pelvic rotation. This protective trunk stiffening strategy implies that Jω may be more sensitive than Jα in postural maintenance tests involving perturbations.

### 3.4. Kinematic Data of the Walking Test

**Figure 6:**
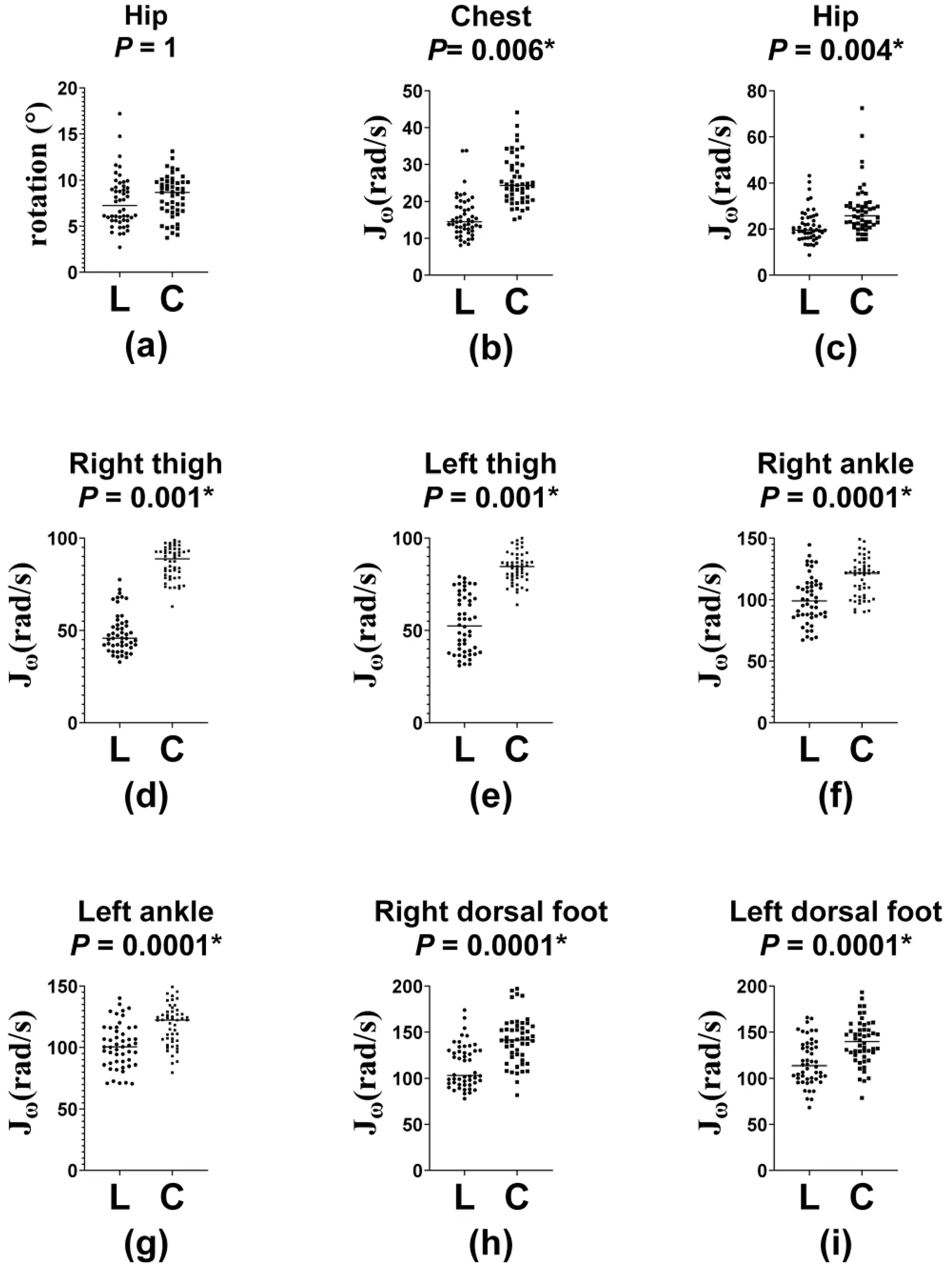
Segmental gait kinematics, including AmpPR (**a**), Jω (**b**–**i**) during normal walking. Data are presented as column scatter plots (horizontal bars represent medians) comparing the chronic low back pain (L) and control (C) cohorts. Significantly reduced Jω  values were observed at the chest (p < 0.001), hip (p  = 0.004), and throughout the thighs, ankles, and feet (all p  < 0.001) in the CLBP group, suggesting an inhibition of rotational activity in the CLBP group during natural walking. * Bonferroni-corrected p-value < 0.05.

During the normal walking test (Figure 6), Jω  exhibited a marked reduction within the CLBP cohort at the chest (p < 0.001), hip (p  = 0.004), and throughout the thighs, ankles, and feet (all p  < 0.001). In contrast, Jα and AmpPR  did not differ significantly between the groups (Figure S4). This selective decline in Jω indicates an inhibition of rotational activity in the CLBP group during natural walking.

**Figure 7:**
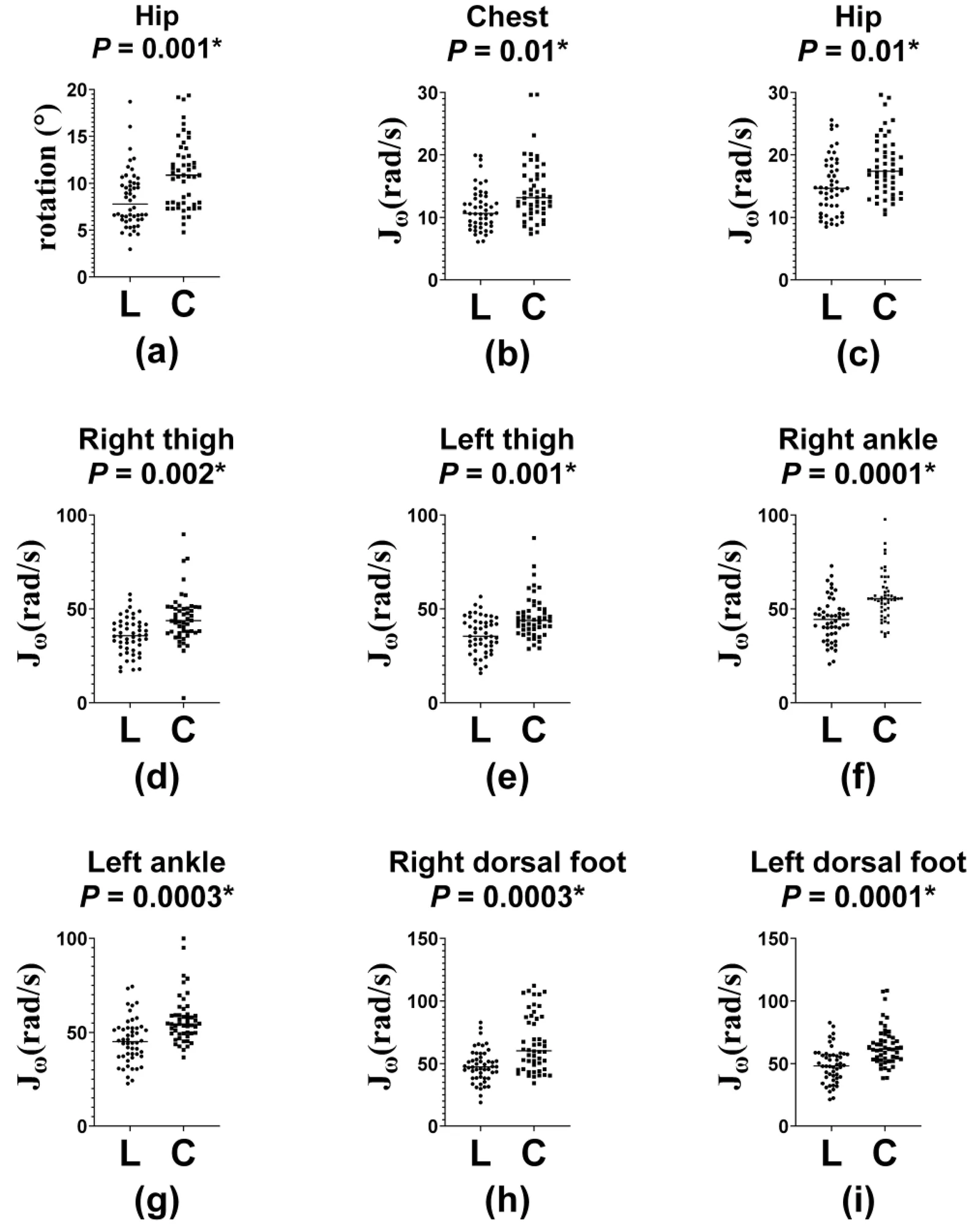
Segmental motion profiles during tandem gait performance, including AmpPR  (**a**) and Jω (**b**–**i**). Data are presented as column scatter plots (horizontal bars represent medians) comparing the chronic low back pain (L) and control (C) cohorts across body segments. Significantly reduced Jω  values were observed across all measured body segments (p < 0.05 for all) in the CLBP group, suggesting an inhibition of rotational activity in the CLBP group during walking with tandem gait. Specifically, Jω diminished substantially among individuals with CLBP (p = 0.001), demonstrating that this cohort tends to automatically limit pelvic rotation to minimize pain during complex walking tasks. * Bonferroni-corrected p-value < 0.05.

During the walking test with tandem gait (Figure 7), higher balance demands were required for dynamic stability. Similar to the findings in the normal walking test, Jω was significantly lower in the CLBP group across all measured body segments (p < 0.05 for all), while Jα still yielded no significant differences (Figure S5). Specifically, AmpPR diminished substantially among individuals with CLBP (p = 0.001). The significant reduction in AmpPR observed only during tandem gait further indicates that the CLBP population may involuntarily restrict pelvic rotations to avoid pain provocation during more challenging tasks.

### 3.5. Kinematic Data of the Five Times Sit-to-Stand Test

**Figure 8:**
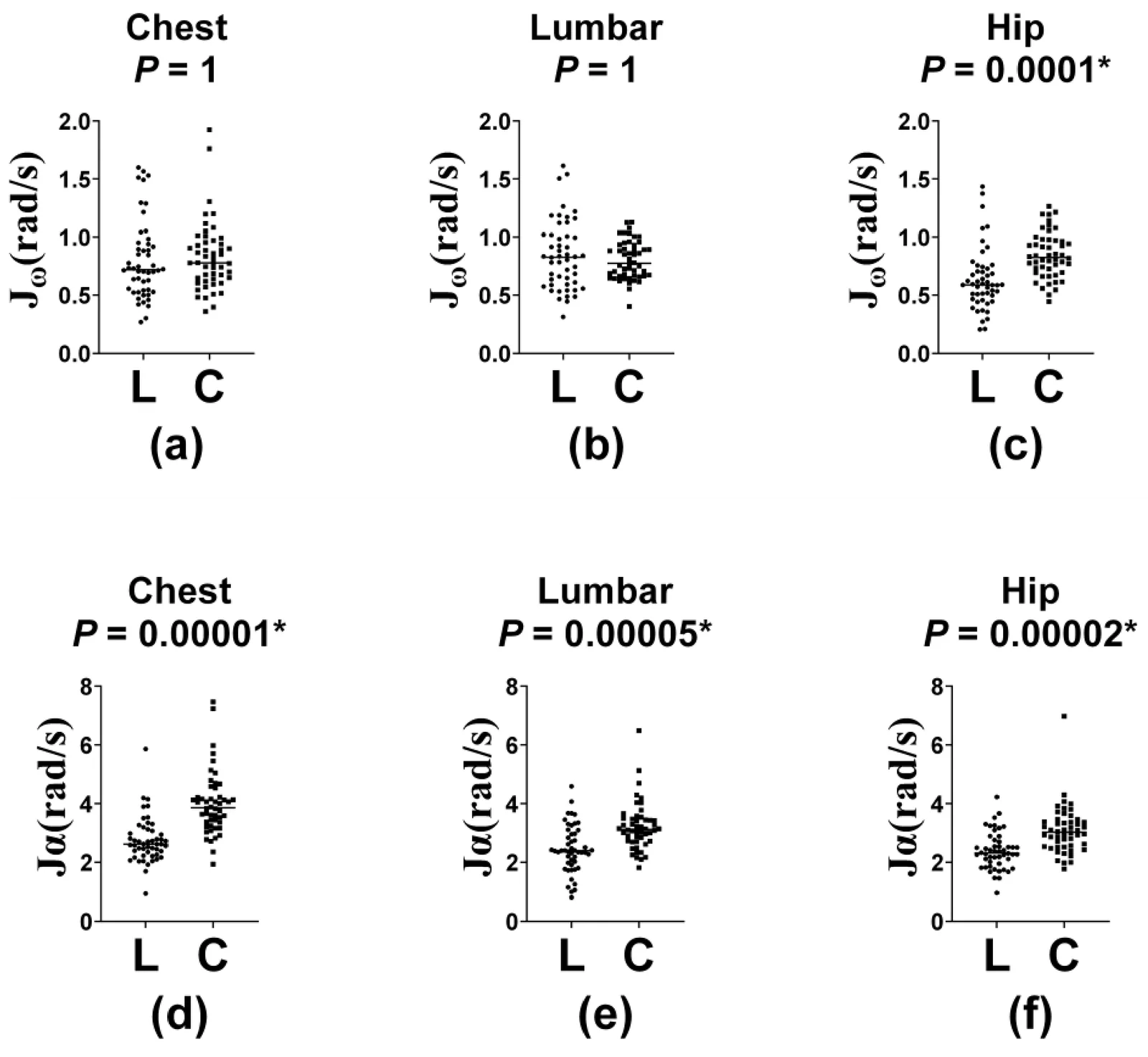
Segmental motion profiles during the sit-to-stand phase of the five times sit-to-stand (FTSS) test, including Jω (**a**–**c**) and  Jα  (**d**–**f**). Data are presented as column scatter plots (horizontal bars represent medians) comparing the chronic low back pain (L) and control (C) cohorts across body segments. In the CLBP group, significantly reduced Jα values were observed across the chest, lumbar spine, and hip (all p < 0.05), with a marked reduction in Jω at the hip (p < 0.05) during the sit-to-stand phase, suggesting a rigid and guarded trunk motion in the CLBP population when rising. * Bonferroni-corrected p-value < 0.05.

To identify specific kinematic impairments at different stages, the FTSS was separated into sit-to-stand (Figure 8 and Figure S6) and stand-to-sit phases (Figure S7). Task completion time was markedly prolonged in the CLBP cohort compared with healthy controls (11.2 ± 4.1 s vs. 9.5 ± 2.0 s; p < 0.001). During the sit-to-stand phase, Jα was significantly lower at the chest, lumbar spine, and hip (all p < 0.05). Furthermore, the hip segment exhibited a marked reduction in Jω within the CLBP group (p < 0.05). In contrast, in the stand-to-sit phase, there were no significant group differences in Jω and Jα across any of the segments.

These results indicate that trunk motion may become rigid and guarded in the CLBP population, particularly when rising. This pattern reinforced the utility of Jω and Jα as outcome measures for clinical assessments and potential interventions for balance dysfunction.

## 4. Discussion

In this study, a wearable IMU system was employed to quantify the balance function of participants with CLBP and healthy controls using specific kinematic indices (Jω, Jα, and AmpPR) under various balance tests. Our results revealed distinct task-dependent kinematic profiles in the CLBP group. While static balance during the SLST showed no significant differences in Jω  or Jα. The introduction of external perturbations during the BBT led to a significant increase in Jω at the chest and hip. These findings point to a compensatory reliance on axial trunk rotation among CLBP cohorts to preserve dynamic balance when facing external perturbations. Conversely, during normal and tandem walking, Jω  decreased significantly across the entire kinetic chain in the CLBP group, with AmpPR showing a significant reduction only during the more challenging tandem gait. During the FTSS, the CLBP group required a longer completion time, driven entirely by the sit-to-stand phase, where Jω at the hip and Jα at the trunk and the hip were significantly lower. Taken together, these findings indicate that individuals with CLBP exhibit a shift in motor strategies based on task demands, utilizing compensatory trunk rotations under perturbation while restoring protective rigidity during self-initiated dynamic transitions.

This study is innovative in that it employs multi-segment evaluation of postural control and gait performance in CLBP, directly aligning with the three main objectives outlined in the introduction. First, multi-site IMU deployment allowed us to identify kinematic differences between the groups and specifically during the rising phase of the FTSS. Second, cross-modal testing revealed task-dependent motor strategies, where patients used compensatory trunk rotations under perturbation (BBT) but adopted protective guarding during walking. Third, these metrics (Jω, Jα, and ${Amp}_{PR}$) demonstrated high sensitivity in distinguishing rotational velocity from linear acceleration changes, detecting functional deficits that standard range of motion measures miss.

The interpretation of Jω and Jα depended on the physical demands of the task. In tasks requiring postural stability, individuals with normal balance control remain mostly stationary. Consequently, frequent postural adjustments typically indicate inferior stability and a reliance on compensatory strategies. Therefore, higher values of Jω and Jα may indicate diminished balance function in tasks designed to maintain a stationary posture. This pattern is consistent with the findings of a systematic review examining the reliability and clinical applicability of IMU-based postural evaluation [52]. In contrast, during dynamic tests, higher Jω and Jα in healthy controls reflected their superior functional mobility and rhythmic coordination. Such biomechanical changes require a distinct perspective when evaluating the CLBP population, where a reduction in Jω and Jα reflected pathological stiffness rather than stability. According to a meta-analysis of walking kinematics in non-specific LBP, the fear of pain could trigger a protective guarding mechanism during locomotion [53]. Consequently, the restricted spinal mobility observed in our CLBP cohort may represent an instinctive compensatory strategy to overcome perceived instability. For these reasons, task-specific patterns require caution when interpreting the results. A recent review emphasized a task-dependent framework to accurately differentiate static instability from dynamic mobility restrictions [54]. Our study incorporated distinct task characteristics, which guided us to further investigate the opposing behavioral patterns between postural maintenance and dynamic functional movements.

The notable contrast between the SLST and BBT outcomes highlights the fact that perturbed tests could reveal subclinical balance deficits in CLBP that simple static tests fail to detect. Although the SLST is widely used in clinical evaluation, our findings show that a firm and fixed surface presents an insufficient sensorimotor challenge. This lack of complexity likely creates a ceiling effect in performance, meaning that the test is not demanding enough to distinguish patients presenting with mild-to-moderate CLBP from asymptomatic controls. On a stable surface, patients can rely on intact distal proprioception, such as ankle strategies, to compensate for the lumbopelvic deficits. This observation aligns closely with the previous literature by MacRae et al., who also reported that people with mild symptoms maintain postural stability comparable to asymptomatic participants during static tasks [55] and Mikkonen et al., who demonstrated that postural sway parameters fail to distinguish patients with CLBP from healthy controls [56]. The lack of statistically meaningful divergence in Jω during the SLST underscores that this test did not place enough postural demand to unmask these hidden differences between groups. Consequently, as observed across the literature, including a systematic review by Zheng et al., CLBP patients can effectively compensate for and conceal their true balance impairments during the SLST [57,58].

In contrast, the BBT introduced self-induced perturbations through its unstable surface, which disrupted these compensatory strategies and exposed the underlying balance deficits. The significant elevation of Jω at the chest and hip indicated that when balance was disturbed by an unstable surface, individuals with CLBP depended more on upper body and pelvic rotations to stabilize themselves. These findings align with the trunk stiffening strategy described by Jones et al., who found that individuals with CLBP tend to co-contract several core muscles to stabilize the spine [8]. This pattern was in accordance with the increased Jω trunk values in the current study. While our study did not directly measure muscle activation, the documented co-contraction phenomenon offers a plausible biomechanical explanation for why our patients appeared to rely on amplified corrective actions from trunk rotation to manage unexpected surface movements. Biomechanically, healthy participants naturally make subtle ankle adjustments to manage minor weight shifts. In contrast, abrupt and compensatory trunk movements were observed in the CLBP group when the center of gravity was lost. This late corrective action prompts the body to perform angular trunk modifications in order to maintain balance.

The discrepancies between the current findings and those of previous studies are likely attributable to differences in experimental paradigms and symptom severity. Although earlier investigations reported greater postural sway in individuals with CLBP during the SLST, these studies often included participants with more pronounced impairment or relied on center of pressure measures, which capture different aspects of postural control from inertial sensor-based measurements [55,57]. Despite these methodological differences, wearable IMUs have demonstrated acceptable reliability for quantifying postural sway, with moderate to good intra-trial consistency (ICC = 0.50–0.67) and good inter-trial consistency (ICC = 0.75–0.86) [59]. On the other hand, our observations closely align with the earlier literature demonstrating that unstable surfaces or semi-tandem stances are more effective in detecting subtle coordination deficits [19]. Corroborating the findings of Alsubaie et al., angular velocity metrics such as Jω appear to be more sensitive than linear acceleration metrics such as Jα under perturbed conditions [60]. This greater sensitivity suggests that balance control under such conditions depends predominantly on continuous rotational adjustments rather than on rigid linear stabilization. Furthermore, previous studies using comparable angular velocity-derived indices from inertial sensors successfully discriminated balance performance across distinct age groups and individuals with knee osteoarthritis [23,30]. Accordingly, the assessment of Jω across multiple body segments during perturbed tasks may offer improved sensitivity for identifying subclinical functional alterations that conventional static measures may fail to detect.

As the balance tests progressed from the static to dynamic challenges, the observed motor control adaptations became more evident. Regarding our testing protocol, arm swing was not restricted during normal walking to maintain natural shoulder and pelvic rotation. Restricting arm motion is known to decrease walking speed and stride length while altering trunk-pelvis movement [61,62,63]. Interestingly, participants with CLBP displayed a global reduction in Jω across the chest, hip, and lower extremities during natural walking with free arm movement, confirming that trunk guarding is a core feature of CLBP. Future research with full-body sensors could compare walking with restricted and free arm swing in the same CLBP cohort to clarify how upper limbs help stabilize the body. Drawing on the previous literature, a plausible hypothesis for this global reduction is that individuals with CLBP unconsciously minimize trunk and pelvic rotations to limit spinal loading and reduce pain provocation [64]. This strategy became particularly apparent during the tandem gait task, which imposed greater balance demands to maintain postural control. During tandem gait, the amplitude of the pelvic rotation was significantly reduced. These findings suggest that individuals with CLBP rely on a protective trunk-stiffening strategy rather than flexible postural adjustments as task demands increase. The findings of the walking test aligned with those of Chen et al., who reported reduced hip and knee range of motion during voluntary weight shifting in individuals with CLBP, further supporting the pattern of reduced segmental mobility seen across tasks [34]. Similarly, Bailes et al. observed that gait variability and compensatory adaptations were more evident in individuals with CLBP and increasing cognitive or balance demands [35]. However, our outcomes challenged earlier reports from Rum et al., who observed increased lumbar transverse displacement and kinematic dispersion during abrupt gait deceleration in CLBP cohorts [65]. This apparent discrepancy is likely attributable to differences in task constraints and control requirements. Steady, continuous walking may favor a rigidity-based guarding strategy to preserve stability, whereas unpredictable gait termination may disrupt motor regulation and elicit the fluctuations observed in their study [66].

Özüdoğru et al. established the FTSS as a robust test for assessing lower extremity neuromuscular integrity and functional independence in CLBP cohorts, providing a useful framework for interpreting the functional alterations observed in the current study [67]. The FTSS results further clarified the functional alterations in guarding strategies. In this study, significant kinematic divergence emerged exclusively during the ascending phase, with no observable inter-group variance during descent. This pattern suggests that rising from a seated position, which requires substantial muscle effort to overcome gravity and transfer body weight, may be particularly sensitive to underlying functional limitations in CLBP. Although previous IMU-based studies reported no meaningful differences in Jα across older adults, individuals with knee osteoarthritis, and yoga practitioners, the current analysis identified distinct differences at the chest and lumbar regions [23,30,31], highlighting the potential utility of Jα for evaluating motor tasks involving rapid physical transitions. We hypothesized that the diminished Jα across the trunk and the hip and Jω at the hip may stem from lumbopelvic muscle weakness and a compromised kinetic chain, though future studies using electromyography or strength testing are needed to confirm this. The marked extension in completion time within the CLBP group likely reflects a protective postural control strategy aimed at minimizing spinal perturbation. Based on established fear-avoidance models, we suggest that this rigid rise is heavily influenced by perceived instability or concerns about pain. Ultimately, our findings indicate that individuals with CLBP display a rigid, guarded movement pattern during dynamic transitions, sacrificing movement efficiency.

These insights offer meaningful clinical utility, informing both functional evaluation strategies and therapeutic intervention design in CLBP management. First, regarding clinical evaluation, our results emphasize that relying solely on static balance assessments such as the SLST may provide a false sense of security regarding a patient’s postural control. Clinicians should use dynamic tasks and unstable surfaces to detect subclinical sensorimotor deficits. Furthermore, wearable IMUs offer sensitive and objective tools for clinicians to quantify specific kinematic alterations in a clinical setting. Second, traditional CLBP programs frequently emphasize core stabilization. However, our data suggest that these patients already employed a rigid protective guard strategy during functional transitions and walking. Therefore, therapeutic interventions should not focus exclusively on core stiffening; rather, they should prioritize dynamic mobility and movement retraining. Treatments aimed at reducing fear avoidance, breaking down protective stiffness, and restoring natural lumbopelvic rotation during walking can significantly improve movement efficiency. Additionally, incorporating perturbation-based balance training could help patients develop better distal reactive strategies (e.g., ankle and hip strategies), reducing their overreliance on exaggerated trunk rotations when encountering unpredictable environments.

Although our findings are supported by the existing literature, several limitations must be acknowledged. First, the study population was intentionally restricted to individuals with mild-to-moderate non-specific CLBP (NPRS 1–6) without prior spinal surgery or neurological comorbidities (e.g., radiculopathy or nerve root compression). While this strict screening minimized confounding structural artifacts and ensured participant safety during high-demand balance and gait tasks, it limits the generalizability of our findings to severe or more complex CLBP cases. Second, motor performance was evaluated solely through IMU-derived kinematics. The absence of electromyography and dynamometry prevented direct measurement of neuromuscular activation patterns and muscle strength. Therefore, conclusions about protective trunk stiffening in gait or lumbopelvic muscle weakness in the rising phase of the FTSS remain biomechanical hypotheses. Third, during the walking tests, participants were not instructed to restrict their arm motion. Natural arm swings may have introduced additional variability into the measurements that our current sensor configuration could not fully isolate or quantify. Fourth, because Jω, Jα, and ${Amp}_{PR}$ represent novel kinematic metrics still under research validation, standardized clinical reference values are currently unavailable. Although we present normative reference values from a healthy control cohort, larger studies including broad age ranges and varying severity levels are required to determine cutoff points for practical clinical application. Finally, the study sample presents limitations in geographic, ethnic, and clinical diversity. Because all participants were recruited from a single regional hospital in Taiwan, the study group is geographically and ethnically homogeneous. In summary, future studies including broader populations, diverse ethnic groups, and different severity levels, while incorporating electromyography and dynamometry, are needed to establish clinical reference ranges for these indices and support their validity as objective biomarkers for balance assessment.

## 5. Conclusions

In this study, multiple IMUs were used to compare balance and gait performance between mild-to-moderate CLBP cohorts and asymptomatic controls. Reflecting the three core objectives of this study, our multi-segment framework demonstrated that CLBP is characterized by task-dependent motor adaptations across functional conditions. Specifically, patients exhibited compensatory trunk rotations (Jω) during perturbed balance on the BBT, protective movement restriction during walking, and reduced linear acceleration (Jα) during the rising phase of the FTSS. These objective metrics allow us to recognize the early balance deficits of patients with CLBP and highlight that rehabilitation should focus on dynamic rotational mobility rather than core stiffening alone. To establish the prognostic value and long-term diagnostic utility of these metrics, expansive longitudinal investigations remain essential.

## Figures and Tables

**Table 1:** IMU positions of different functional tests.

Balance board test	Chest, hip, ^1^ knees/ankles, R. dorsal foot, balance board
Walking test	Chest, hip, ^1^ thighs/ankles/dorsal feet
Single-leg stance test	Chest, lumbar, hip, ^1^ thighs/ankles, standing dorsal foot
Five times sit-to-stand test	Chest, lumbar, hip, ^1^ thighs

^1^ Bilateral.

**Table 2:** Demographic data.

Characteristics	^1^ CLBP Group	Control Group	*p*-Value
Number, n	50	50	-
Age, years	65.68 ± 11.84	65.26 ± 10.73	0.85
Female, n (%)	36 (72%)	32 (64%)	-
Body mass index	24.86 ± 4.01	25.17 ± 5.23	0.74
Numeric pain rating scale	4.38 ± 1.47	-	-
Back pain duration, years	6.94 ± 5.52	-	-
Roland–Morris Questionnaire, points	7.14 ± 4.05	-	-

^1^ CLBP: chronic low back pain; data are number (%) or mean ± SD.

## Data Availability

Due to institutional privacy regulations and ethical constraints, raw patient data are not publicly shared. Fully de-identified datasets will be made available upon reasonable request to the corresponding author, subject to prior Institutional Review Board clearance and a formal data use agreement.

## Supplementary Materials

The following supporting information can be downloaded at: https://www.mdpi.com/article/10.3390/s26196182/s1, Figure S1: Segmental distribution of kinematic data in single-leg stance test; Figure S2: Segmental distribution of kinematic data in balance board test, stances with shoulder-width; Figure S3: Segmental distribution of kinematic data in balance board test, stances with feet-together; Figure S4: Segmental distribution of kinematic data in normal walking test; Figure S5: Segmental distribution of kinematic data in walking test with tandem gait; Figure S6: Segmental distribution of kinematic data in five time sit-to-stand test, sit-to-stand phase; Figure S7: Segmental distribution of kinematic data in five time sit-to-stand test, stand-to-sit phase; Table S1: Kinematic data.

## Abbreviations

CLBP	Chronic low back pain
IMU	Inertial measurement units
Jω	Average absolute angular velocity
Jα	Average absolute linear acceleration
TUG	Timed up and go
SLST	Single-leg stance test
FTSS	Five times sit-to-stand
MAD	Mean amplitude deviation
${Amp}_{PR}$	Pelvic rotation amplitude
BBT	Balance board test
NPRS	Numeric pain rating scale
RMDQ	Roland–Morris Disability Questionnaire
MANOVA	Multivariate Analysis of Variance
SD	Standard deviation
s	Seconds

## Appendix A

Detailed specifications for the IMU system are accessible online at https://reurl.cc/8YNQOg (accessed on 19 August 2026).
