# Supplementary material for: Assessment of Task-Specific Gait and Balance Deficits in Chronic Low Back Pain Using Multi-Sensor Inertial Measurement Units
Source: Sensors (Basel). 2026 Sep 29;26(19):6182. doi: 10.3390/s26196182 (PMC13667449; doi:10.3390/s26196182)
Supplement: Supplementary file 1 [file sensors-26-06182-s001.zip › sensors-4557842-supplementary.pdf]

Supplementary Materials (Figure S1-S7; Table S1)

Segmental distribution of kinematic data (Figure S1-S7)

Figure S1. Single-Leg Stance Test

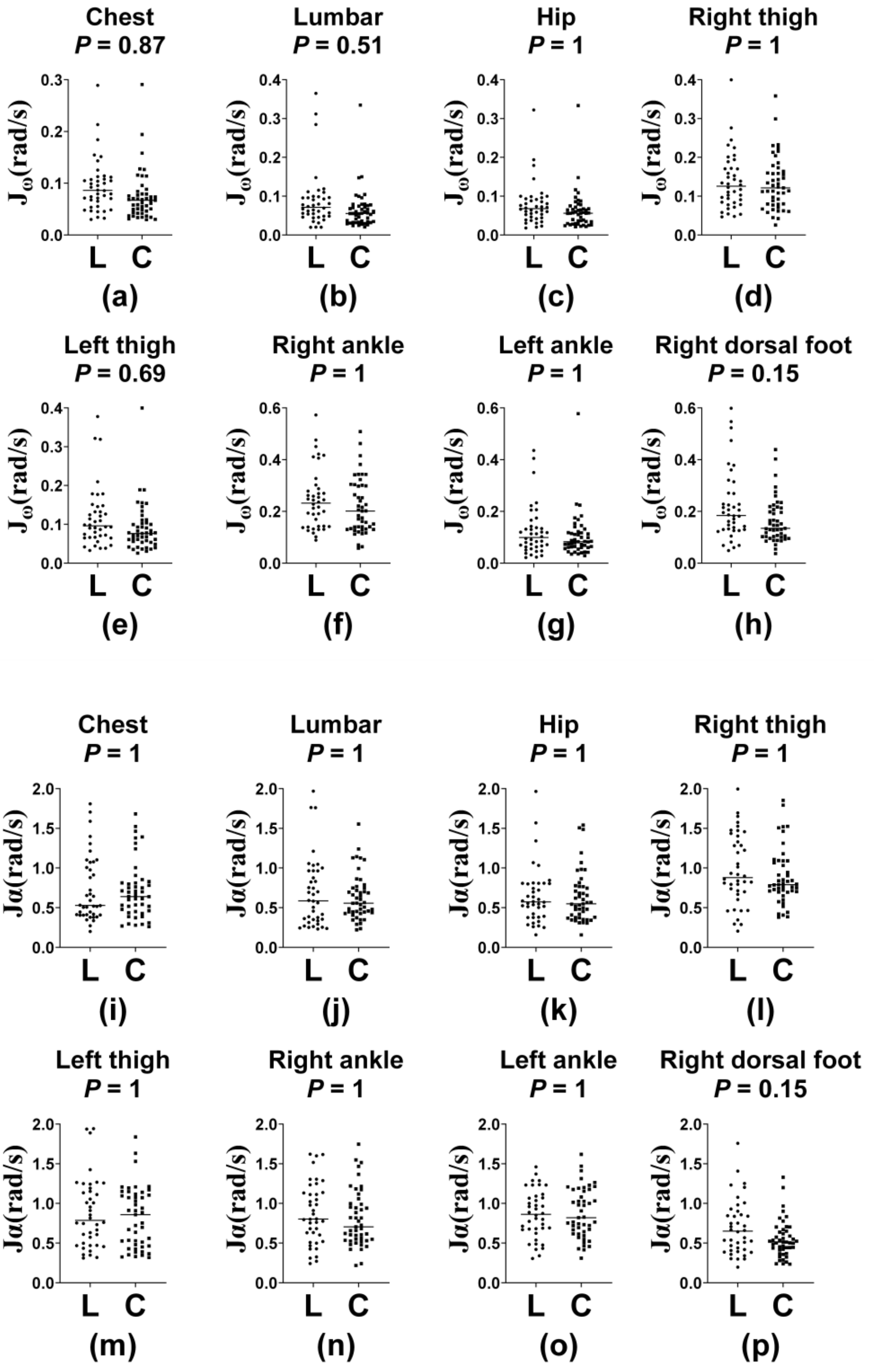

Figure S1. Segmental distribution of $J\omega$ (a—h) and $J\alpha$ (i—p) across body segments during the Single-Leg Stance test. Data are presented as column scatter plots (horizontal bars indicate medians) comparing the chronic low back pain (L) and control (C) groups across body segments. P: Bonferroni-corrected p-value.

Figure S2. Balance board test, stances with shoulder-width

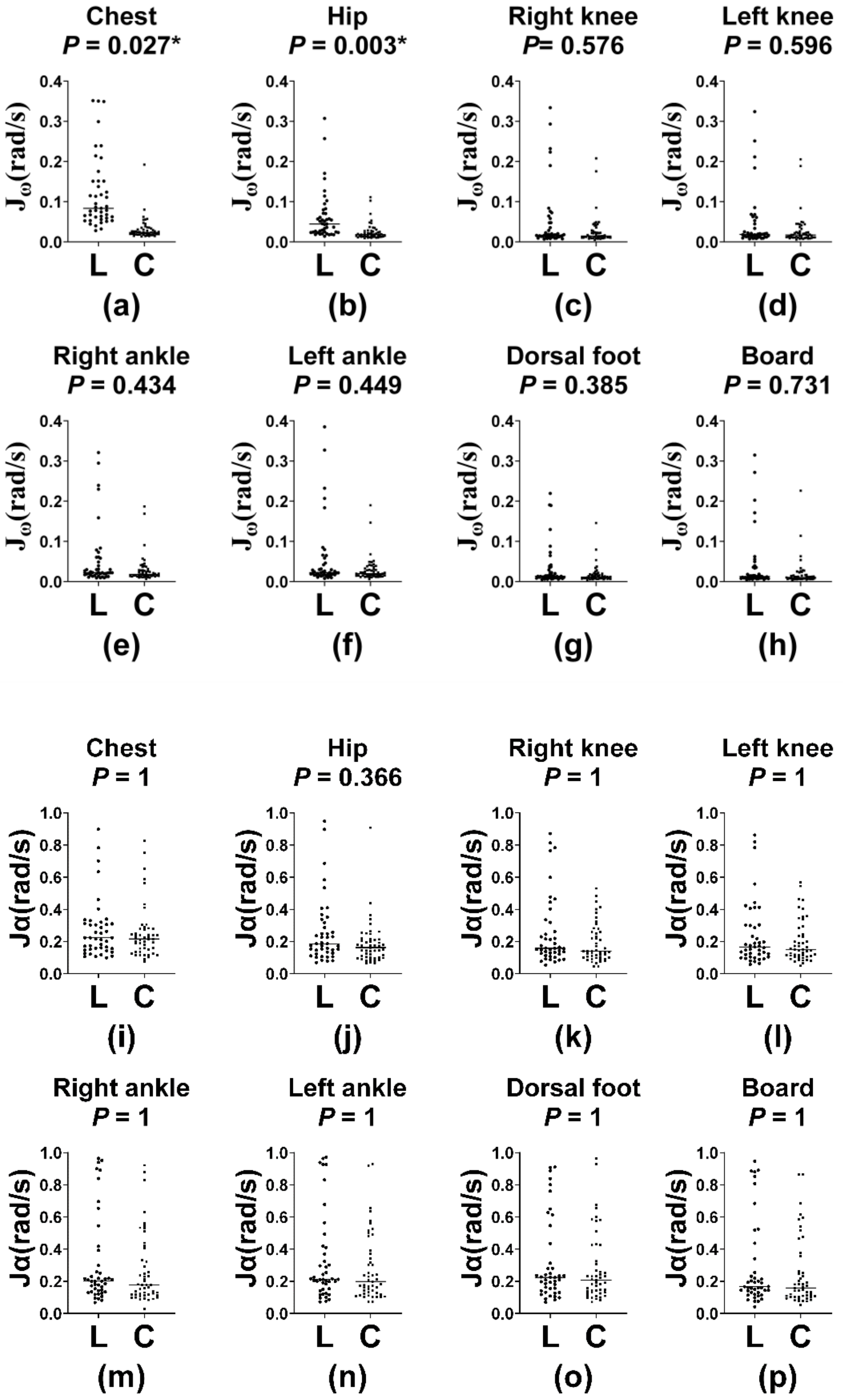

Figure S2. Segmental distribution of $J\omega$ (a—h) and $J\alpha$ (i—p) across body segments during the Balance Board stances with shoulder-width. Data are presented as column scatter plots (horizontal bars indicate medians) comparing the chronic low back pain (L) and control (C) groups across body segments. * Bonferroni-corrected p-value < 0.05.

Figure S3. Balance board test, stances with feet-together

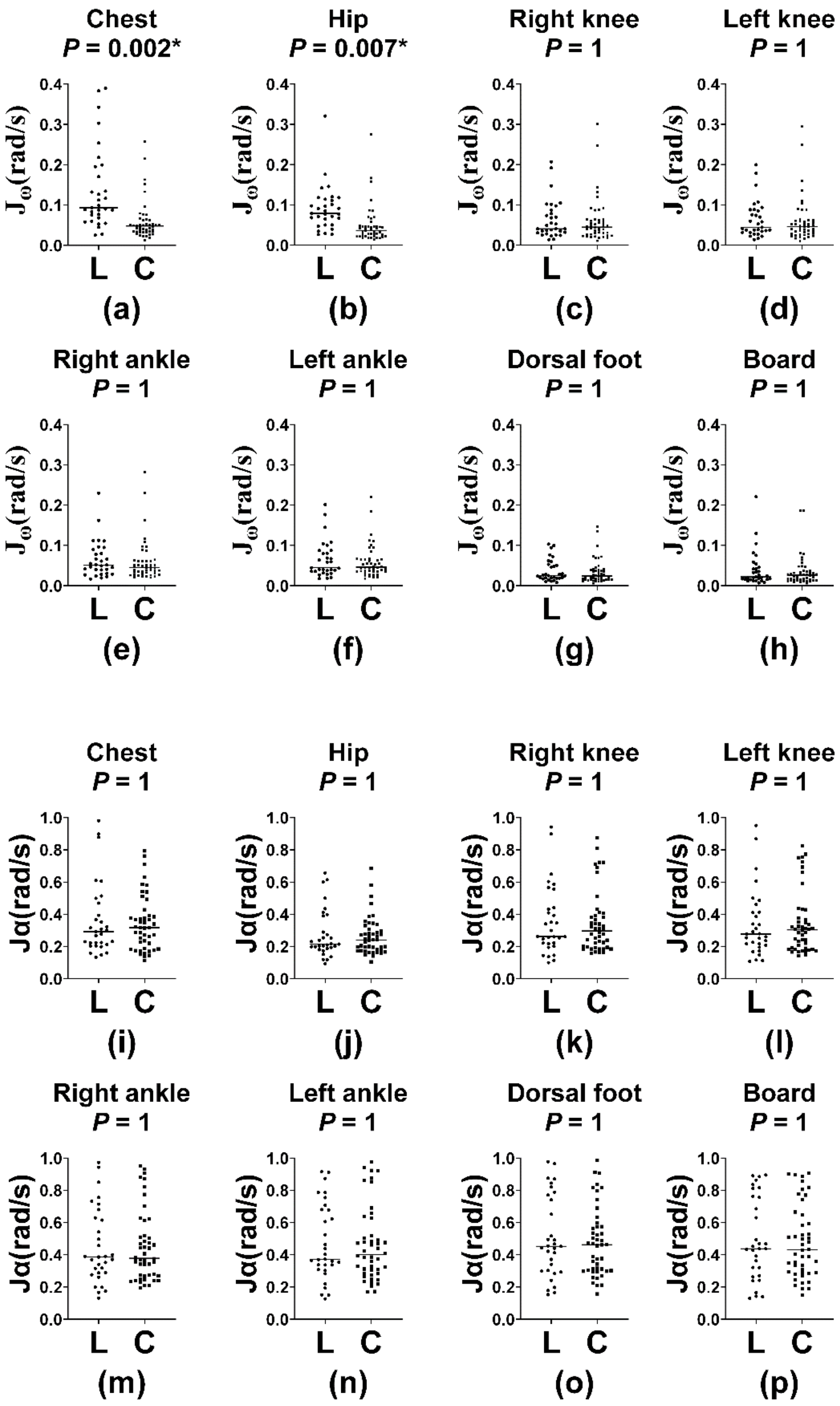

Figure S3. Segmental distribution of $J\omega$ (a—h) and $J\alpha$ (i—p) across body segments during the Balance Board stances with feet-together. Data are presented as column scatter plots (horizontal bars indicate medians) comparing the chronic low back pain (L) and control (C) groups across body segments. * Bonferroni-corrected p-value < 0.05.

Figure S4. Normal walking test

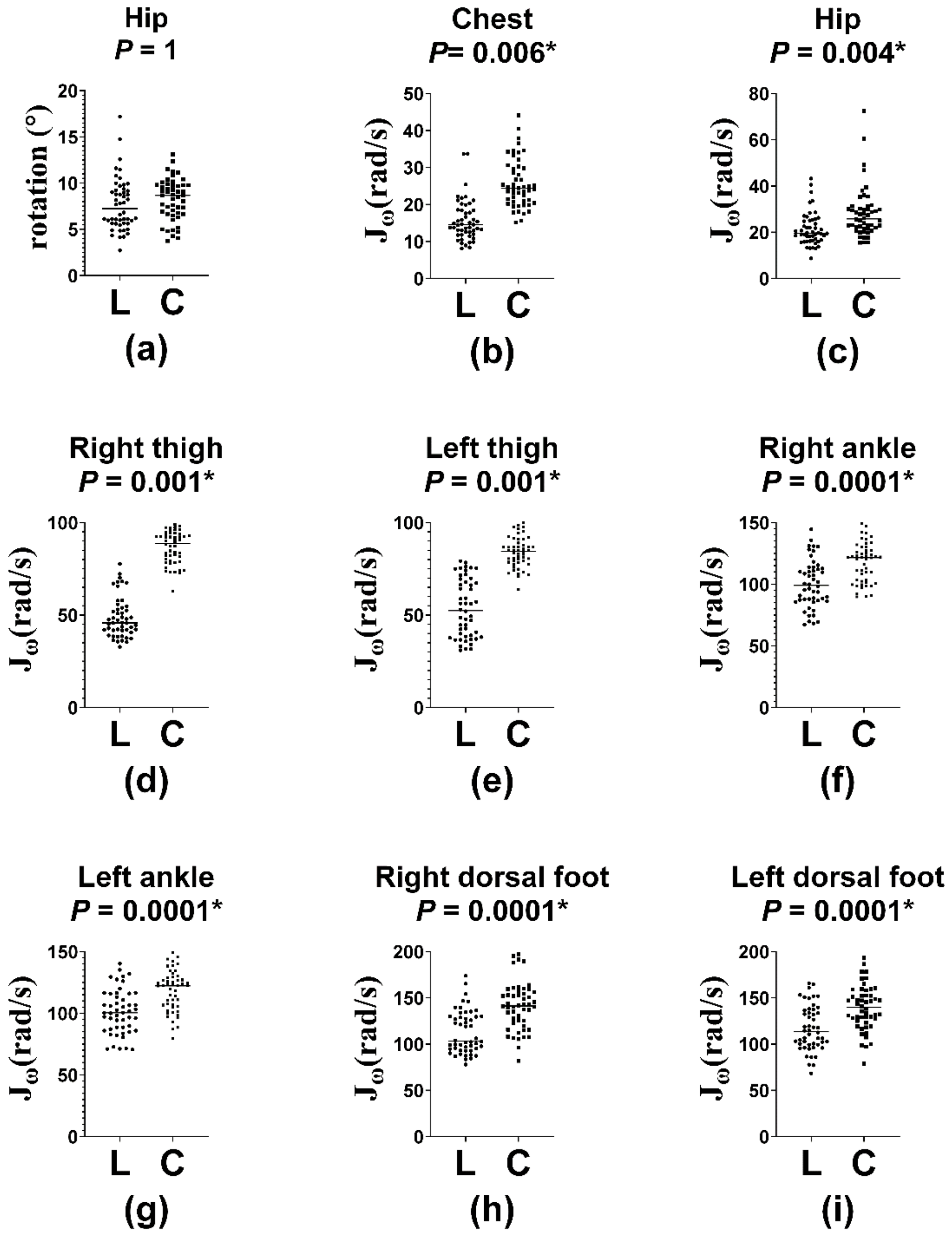

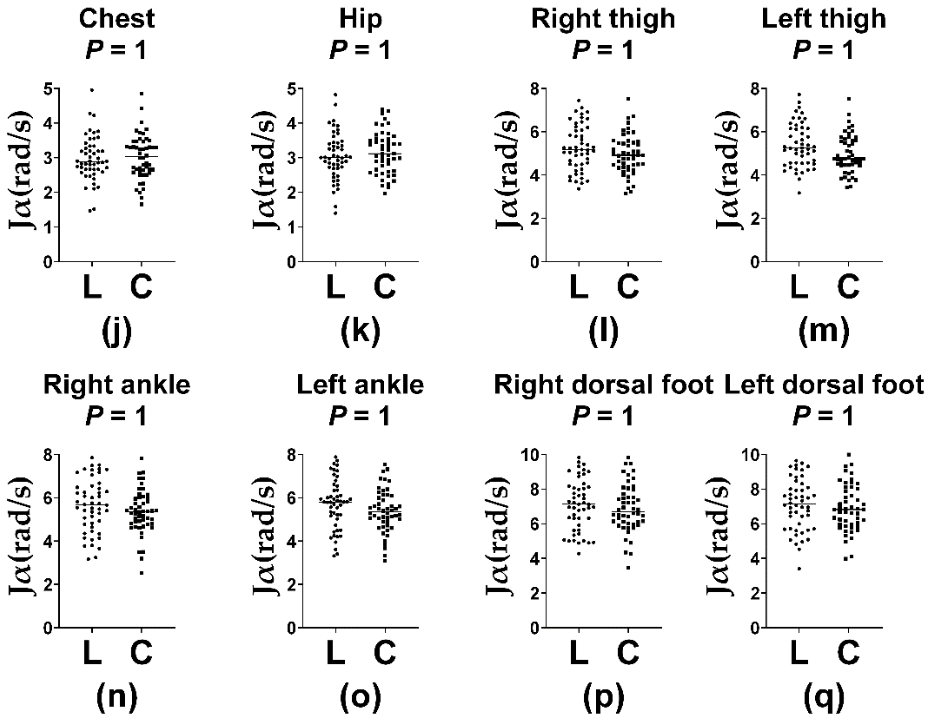

Figure S4. Segmental gait kinematics including $AmpPR$(a), $J\omega$ (b—i), and $J\alpha$ (j—q) during normal walking. Data are presented as column scatter plots (horizontal bars represent medians) comparing the chronic low back pain (L) and control (C) cohorts. * Bonferroni-corrected p-value < 0.05.

Figure S5. Walking test with tandem gait

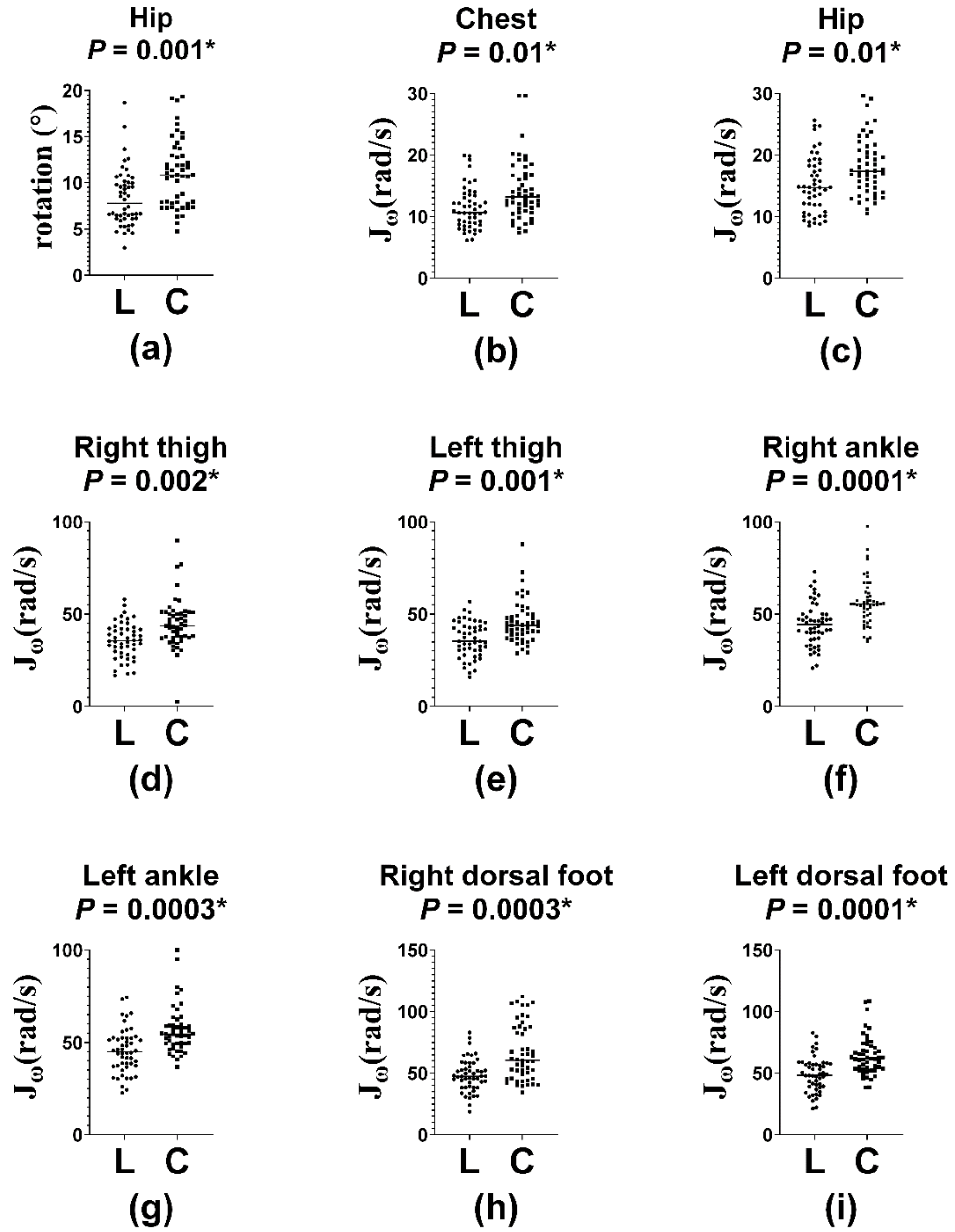

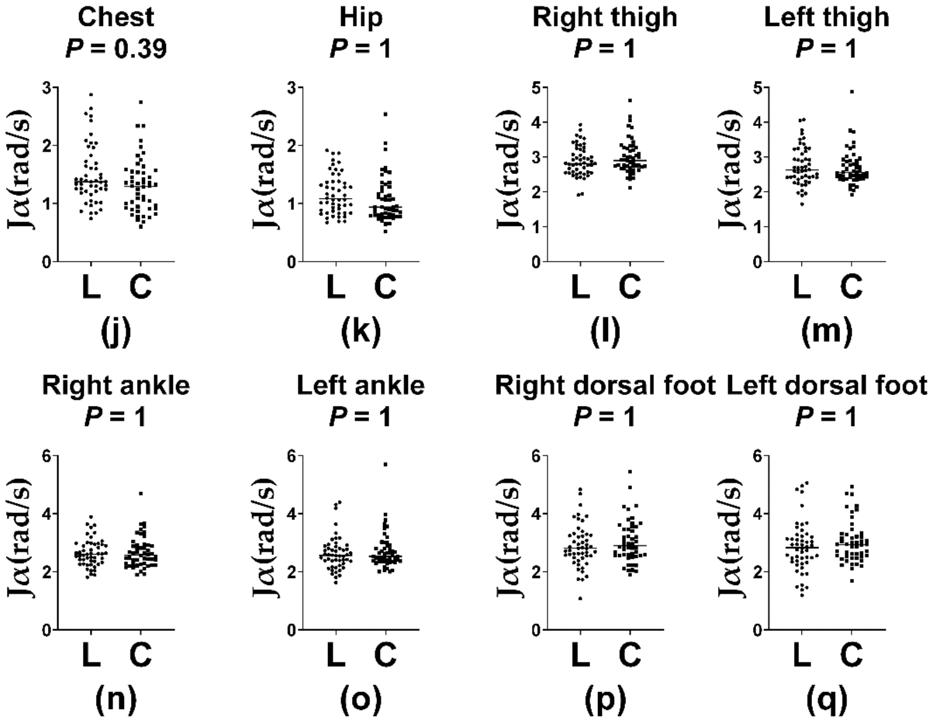

Figure S5. Segmental motion profiles during tandem gait performance, including $AmpPR$ (a), $J\omega$ (b—i), and $J\alpha$ (j—q). Data are presented as column scatter plots (horizontal bars represent medians) comparing the chronic low back pain (L) and control (C) cohorts across body segments. * Bonferroni-corrected p-value < 0.05.

Figure S6. Five time sit-to-stand test, sit-to-stand phase

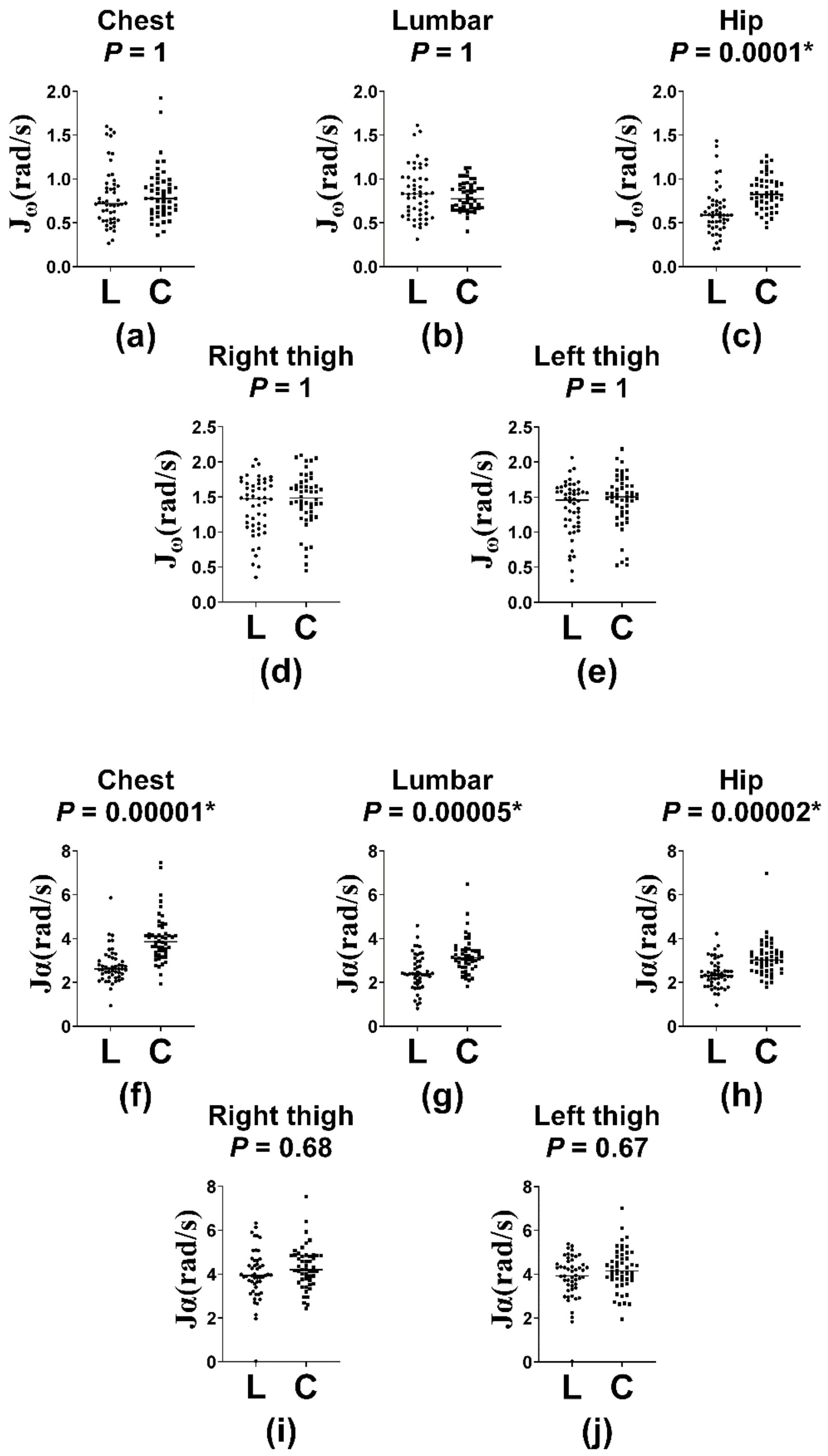

Figure S6. Segmental motion profiles during the sit-to-stand phase of the Five Times Sit-to-Stand (FTSS) test, including $J\omega$ (a—e) and $J\alpha$ (f—j). Data are presented as column scatter plots (horizontal bars represent medians) comparing the chronic low back pain (L) and control (C) cohorts across body segments. * Bonferroni-corrected p-value < 0.05.

Figure S7. Five time sit-to-stand test, stand-to-sit phase

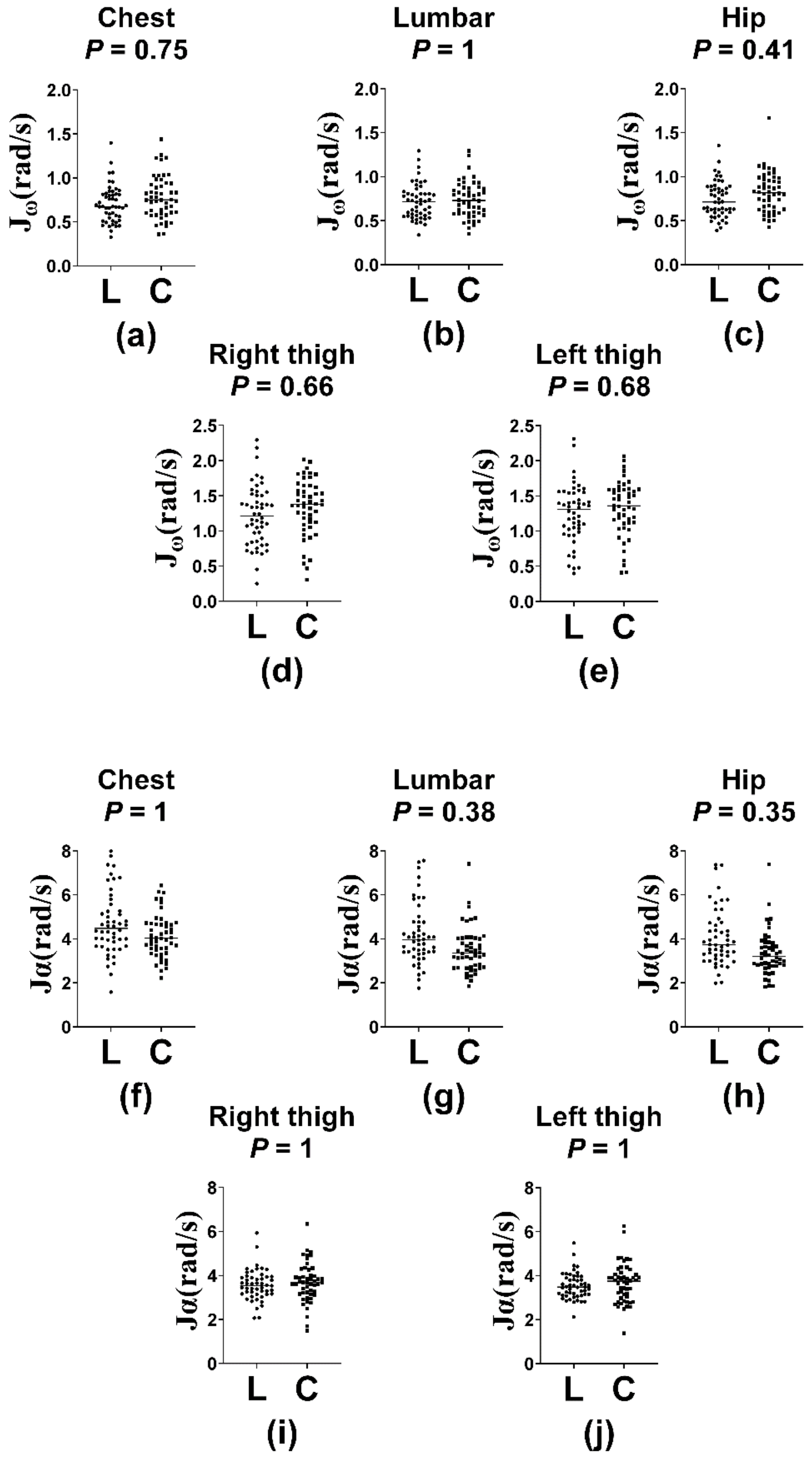

Figure S7 Segmental motion profiles during the stand-to-sit phase of the Five Times Sit-to-Stand (FTSS) test, including $J\omega$ (a—e) and $J\alpha$ (f—j). Data are presented as column scatter plots (horizontal bars represent medians) comparing the chronic low back pain (L) and control (C) cohorts across body segments.

Table S1 - Kinematic data

$J\omega$ in Single-Leg Stance Test (rad/s)

Location	CLBP Group	Control Group
Chest	0.097 ± 0.062	0.076 ± 0.058
Lumbar	0.089 ± 0.073	0.064 ± 0.050
Hip	0.078 ± 0.055	0.062 ± 0.049
Stance-limb thigh	0.137 ± 0.073	0.136 ± 0.087
Contralateral thigh	0.127 ± 0.114	0.092 ± 0.073
Stance-limb ankle	0.244 ± 0.115	0.218 ± 0.107
Contralateral ankle	0.124 ± 0.096	0.104 ± 0.085
Stance dorsal foot	0.221 ± 0.137	0.163 ± 0.086

CLBP: chronic low back pain ; Data are mean ± SD.

$J\alpha$ in Single-Leg Stance Test (rad/s)

Location	CLBP Group	Control Group
Chest	0.730 ± 0.418	0.710 ± 0.358
Lumbar	0.699 ± 0.475	0.631 ± 0.288
Hip	0.673 ± 0.472	0.656 ± 0.405
Stance-limb thigh	1.049 ± 0.763	0.886 ± 0.354
Contralateral thigh	0.882 ± 2.886	0.823 ± 2.848
Stance-limb ankle	0.874 ± 0.387	0.807 ± 0.354
Contralateral ankle	0.862 ± 2.418	0.868 ± 2.829
Stance dorsal foot	0.705 ± 0.373	0.547 ± 0.232

CLBP: chronic low back pain ; Data are mean ± SD.

$J\omega$ in Balance Board Test, stances with shoulder-width (rad/s)

Location	CLBP Group	Control Group
Chest	0.183 ± 0.352	0.031 ± 0.027
Hip	0.065 ± 0.072	0.025 ± 0.021
Right knee	0.056 ± 0.099	0.028 ± 0.038
Left knee	0.056 ± 0.099	0.029 ± 0.038
Right ankle	0.059 ± 0.096	0.030 ± 0.034
Left ankle	0.057 ± 0.091	0.030 ± 0.032
Dorsal foot	0.040 ± 0.074	0.018 ± 0.023
Board	0.048 ± 0.105	0.020 ± 0.035

CLBP: chronic low back pain ; Data are mean ± SD.

$J\alpha$ in Balance Board Test, stances with shoulder-width (rad/s)

Location	CLBP Group	Control Group
Chest	0.278 ± 0.210	0.320 ± 0.531
Hip	0.261 ± 0.221	0.185 ± 0.133
Right knee	0.284 ± 0.348	0.210 ± 0.223
Left knee	0.278 ± 0.319	0.221 ± 0.230
Right ankle	0.397 ± 0.543	0.298 ± 0.359

Left ankle	0.408 ± 0.530	0.307 ± 0.337
Dorsal foot	0.442 ± 0.627	0.318 ± 0.389
Board	0.411 ± 0.621	0.284 ± 0.373

CLBP: chronic low back pain ; Data are mean ± SD.

$J\omega$ in Balance Board Test, stances with feet-together (rad/s)

Location	CLBP Group	Control Group
Chest	0.157 ± 0.160	0.060 ± 0.049
Hip	0.091 ± 0.056	0.050 ± 0.047
Right knee	0.064 ± 0.048	0.060 ± 0.055
Left knee	0.064 ± 0.046	0.060 ± 0.055
Right ankle	0.065 ± 0.046	0.060 ± 0.052
Left ankle	0.065 ± 0.045	0.059 ± 0.041
Dorsal foot	0.038 ± 0.028	0.035 ± 0.030
Board	0.040 ± 0.044	0.035 ± 0.037

CLBP: chronic low back pain ; Data are mean ± SD.

$J\alpha$ in Balance Board Test, stances with feet-together (rad/s)

Location	CLBP Group	Control Group
Chest	0.361 ± 0.225	0.365 ± 0.221
Hip	0.273 ± 0.154	0.261 ± 0.122
Right knee	0.360 ± 0.223	0.369 ± 0.270
Left knee	0.339 ± 0.207	0.366 ± 0.280
Right ankle	0.516 ± 0.354	0.520 ± 0.400
Left ankle	0.530 ± 0.353	0.538 ± 0.423
Dorsal foot	0.578 ± 0.402	0.609 ± 0.469
Board	0.582 ± 0.418	0.598 ± 0.508

CLBP: chronic low back pain ; Data are mean ± SD.

Amp_{PR} in Normal Walking Test (degrees)

	CLBP Group	Control Group
Pelvic rotation	7.678 ± 2.839	8.241 ± 2.249

CLBP: chronic low back pain ; Data are mean ± SD.

$J\omega$ in Normal Walking Test (rad/s)

Location	CLBP Group	Control Group
Chest	15.678 ± 5.526	25.659 ± 6.582
Hip	21.353 ± 7.079	28.348 ± 11.710
Right thigh	76.376 ± 19.467	90.547 ± 18.053
Left thigh	78.626 ± 19.344	93.988 ± 18.844
Right ankle	99.185 ± 21.287	117.409 ± 17.668
Left ankle	100.253 ± 19.974	118.444 ± 17.319
Right dorsal foot	116.552 ± 27.616	141.393 ± 26.740
Left dorsal foot	116.004 ± 26.678	139.234 ± 24.036

CLBP: chronic low back pain ; Data are mean ± SD.

$J\alpha$ in Normal Walking Test (rad/s)

Location	CLBP Group	Control Group
Chest	2.972 ± 0.647	3.019 ± 0.641
Hip	3.048 ± 0.691	3.166 ± 0.663
Right thigh	5.237 ± 1.108	4.946 ± 0.925
Left thigh	5.360 ± 1.068	4.977 ± 0.886
Right ankle	5.641 ± 1.312	5.349 ± 1.024
Left ankle	5.720 ± 1.280	5.392 ± 0.988
Right dorsal foot	7.072 ± 1.722	6.882 ± 1.384
Left dorsal foot	7.165 ± 1.705	6.911 ± 1.350

CLBP: chronic low back pain ; Data are mean ± SD.

Amp_{PR} in Tandem Gait Walking Test (degrees)

	CLBP Group	Control Group
Pelvic rotation	8.343 ± 3.086	11.067 ± 3.827

CLBP: chronic low back pain ; Data are mean ± SD.

$J\omega$ in Tandem Gait Walking Test (rad/s)

Location	CLBP Group	Control Group
Chest	11.358 ± 3.427	14.253 ± 5.024
Hip	15.088 ± 4.661	18.216 ± 4.921
Right thigh	35.626 ± 9.705	45.303 ± 15.060
Left thigh	36.037 ± 9.635	45.220 ± 13.565
Right ankle	44.319 ± 11.644	57.175 ± 14.991
Left ankle	45.412 ± 12.020	57.298 ± 15.144
Right dorsal foot	48.191 ± 13.224	62.752 ± 19.519
Left dorsal foot	48.891 ± 14.113	65.363 ± 21.591

CLBP: chronic low back pain ; Data are mean ± SD.

$J\alpha$ in Tandem Gait Walking Test (rad/s)

Location	CLBP Group	Control Group
Chest	1.506 ± 0.518	1.309 ± 0.467
Hip	1.164 ± 0.345	1.070 ± 0.395
Right thigh	2.890 ± 0.434	3.024 ± 0.577
Left thigh	2.741 ± 0.556	2.696 ± 0.526
Right ankle	2.649 ± 0.469	2.695 ± 0.627
Left ankle	2.635 ± 0.586	2.715 ± 0.659
Right dorsal foot	2.888 ± 0.742	3.127 ± 1.017
Left dorsal foot	2.858 ± 0.884	3.123 ± 1.003

CLBP: chronic low back pain ; Data are mean ± SD.

$J\omega$ in Five Times Sit-to-Stand Test, sit-to-stand phase (rad/s)

Location	CLBP Group	Control Group
Chest	0.798 ± 0.339	0.930 ± 0.901
Lumbar	0.843 ± 0.297	0.801 ± 0.164
Hip	0.633 ± 0.268	0.843 ± 0.196
Right thigh	1.359 ± 0.434	1.457 ± 0.388
Left thigh	1.330 ± 0.419	1.448 ± 0.378

CLBP: chronic low back pain ; Data are mean ± SD.

$J\alpha$ in Five Times Sit-to-Stand Test, sit-to-stand phase (rad/s)

Location	CLBP Group	Control Group
Chest	2.744 ± 0.769	4.151 ± 1.885
Lumbar	2.459 ± 0.804	3.205 ± 0.809
Hip	2.389 ± 0.627	3.086 ± 0.811
Right thigh	3.984 ± 1.136	4.299 ± 0.955
Left thigh	3.864 ± 0.997	4.212 ± 0.961

CLBP: chronic low back pain ; Data are mean ± SD.

$J\omega$ in Five Times Sit-to-Stand Test, stand-to-sit phase (rad/s)

Location	CLBP Group	Control Group
Chest	0.705 ± 0.210	0.962 ± 1.234
Lumbar	0.718 ± 0.200	0.738 ± 0.203
Hip	0.745 ± 0.206	0.821 ± 0.226
Right thigh	1.204 ± 0.460	1.335 ± 0.399
Left thigh	1.228 ± 0.461	1.322 ± 0.389

CLBP: chronic low back pain ; Data are mean ± SD.

$J\alpha$ in Five Times Sit-to-Stand Test, stand-to-sit phase (rad/s)

Location	CLBP Group	Control Group
Chest	4.851 ± 1.884	4.531 ± 2.997
Lumbar	4.296 ± 1.672	3.655 ± 1.901
Hip	4.145 ± 1.560	3.511 ± 1.894
Right thigh	3.468 ± 0.926	3.675 ± 0.885
Left thigh	3.430 ± 0.933	3.636 ± 0.925

CLBP: chronic low back pain ; Data are mean ± SD.
